# Longitudinal and adjusted prognostic assessment of calprotectin in Gram-negative sepsis with acute respiratory failure

**DOI:** 10.1128/spectrum.01767-26

**Published:** 2026-09-15

**Authors:** Simona Gigliotti, Michele Manno, Manuela Colosimo, Francesca Divenuto, Francesco Dragone, Marta Greco, Cinzia Peronace, Luigia Gallo, Francesca Trimboli, Pasquale Minchella, Francesca Greco, Alba Silipo, Nadia Marascio, Eugenio Garofalo, Federico Longhini, Angela Quirino, Giovanni Matera

**Affiliations:** 1 Clinical Microbiology Unit, Department of Health Sciences, Magna Graecia University 548460, Catanzaro, Italy; 2 Microbiology and Virology Unit, R. Dulbecco University Hospital 220603 , Catanzaro, Italy; 3 Clinical Pathology Unit, Department of Health Sciences, University Magna Græcia of Catanzaro 548460, Catanzaro, Italy; 4 Clinical Microbiology Unit, Renato Dulbecco University Hospital 220603 , Catanzaro, Italy; 5 Microbiology Unit, Annunziata Hub Hospital, Azienda Ospedaliera di Cosenza 220599, Cosenza, Italy; 6 Department of Chemical Sciences, University of Naples Federico II 9307, Naples, Italy; 7 Anesthesia and Intensive Care, Department of Medical and Surgical Sciences, Magna Graecia University 335566, Catanzaro, Italy; Instituto de Salud Global de Barcelona, Barcelona, Spain

**Keywords:** sepsis, calprotectin, biomarkers, cytokines, inflammation, mortality

## Abstract

Sepsis caused by Gram-negative bloodstream infection is characterized by dynamic host responses, but the longitudinal and incremental prognostic value of calprotectin remains uncertain. We conducted a multicenter retrospective observational study of 82 adults with Gram-negative bloodstream infection, sepsis, and acute respiratory failure. Serum calprotectin, routine biomarkers, cytokines, and growth factors were measured at diagnosis before antibiotic therapy (T0), day 3 (T1), and day 7 (T2), and compared according to 28-day survival. Sixty-four patients survived, and 18 died. Calprotectin declined over time but remained higher in non-survivors at T0 (*P* = 0.003), T1 (*P* = 0.037), and T2 (*P* = 0.035); its longitudinal trajectory did not differ between survivors and non-survivors (*P* = 0.408). Selected routine and immune biomarkers showed nominal differences at T2, but none of the 63 secondary biomarker-by-time comparisons remained significant after false-discovery-rate correction. Sequential organ failure assessment 2 (SOFA-2) alone strongly discriminated mortality (area under the receiver operating characteristic curve [AUC], 0.897; 95% CI, 0.828–0.965) and outperformed baseline calprotectin (AUC, 0.728; DeLong *P* = 0.027). Baseline calprotectin remained independently associated with mortality after adjustment for age and SOFA-2 (OR, 2.07 per doubling; 95% CI, 1.14–4.20; *P* = 0.018), but its addition yielded only a modest, non-significant improvement in discrimination (ΔAUC, 0.023; *P* = 0.117). SOFA-2 was the dominant prognostic discriminator; calprotectin may provide complementary rather than standalone prognostic information and requires external validation.

**IMPORTANCE:**

Gram-negative bloodstream infection accompanied by acute respiratory failure carries a substantial risk of death. In this cohort, sequential organ failure assessment 2 (SOFA-2) was the strongest early predictor of 28-day mortality. Calprotectin was higher in non-survivors from diagnosis through day 7 and remained associated with mortality after adjustment for age and SOFA-2, but adding it to the clinical model produced only a modest and statistically non-significant improvement in discrimination. Other routine and immune biomarkers showed nominal late differences that did not remain significant after false-discovery-rate correction. These findings suggest that calprotectin may complement, but should not replace, established clinical severity assessment. Larger prospective cohorts with external validation are required before calprotectin or multimarker panels can be used for clinical risk stratification.

## INTRODUCTION

Sepsis is a heterogeneous and time-dependent syndrome defined as life-threatening organ dysfunction caused by a dysregulated host response to infection (1). Despite advances in early recognition, source control, antimicrobial therapy, and organ support, it remains a major global health burden with high morbidity and mortality, particularly in critically ill patients and in those with bloodstream infection and organ failure (2, 3). Its course reflects the evolving interplay between pathogen-related factors and host responses, including inflammatory activation, endothelial injury, coagulation abnormalities, microcirculatory dysfunction, and immune dysregulation.

Because sepsis evolves over time, its biological characterization cannot rely on single-point measurements alone. Serial biomarker assessment may better capture the trajectory of the host response, treatment effectiveness, and risk of adverse outcomes; therefore, biomarker kinetics may be more informative than isolated baseline values (4). However, most studies have focused on diagnostic performance or baseline prognostic value, whereas fewer have explored biomarker behavior longitudinally during acute sepsis (5–7).

Among circulating inflammatory markers, calprotectin has emerged as a promising candidate. Calprotectin, the S100A8/S100A9 heterodimer, is highly abundant in neutrophils and is released during innate immune activation, thereby reflecting neutrophil-driven inflammation (8–10). Given the central role of neutrophils in sepsis pathophysiology, calprotectin may provide biologically relevant information beyond conventional inflammatory markers. Previous studies have suggested diagnostic and prognostic value for circulating calprotectin in sepsis (11–14), but data on its temporal profile remain limited. The few available longitudinal studies were conducted either in postoperative ICU patients or in broader critically ill septic populations, with different sampling schedules and clinical settings (13, 15, 16). In a previous study, we investigated the diagnostic and prognostic role of neutrophil-related biomarkers in sepsis (8). Building on that work, we hypothesized that serial calprotectin measurements would provide clinically relevant information on disease evolution and outcome, and that its longitudinal trajectory might differ between survivors and non-survivors. Accordingly, in this multicenter retrospective observational study, we sought to characterize the longitudinal profile of circulating calprotectin across three prespecified time points in a clinically well-defined cohort of patients with sepsis due to Gram-negative bloodstream infection and concomitant acute respiratory failure (ARF). We further aimed to determine whether calprotectin differed according to 28-day mortality, both in terms of absolute concentrations at individual time points and temporal trajectories over time, and to contextualize these findings through parallel assessment of routine biomarkers, cytokines, and growth factors. Finally, for biomarkers showing outcome-related differences, we explored their ability to discriminate 28-day mortality.

## MATERIALS AND METHODS

The study protocol was approved by the Ethics Committee of the Calabria Region (approval no. 128/2023, 22 December 2023). Given the retrospective design of the study, the requirement for written informed consent was waived. All procedures were performed in accordance with the principles of the Declaration of Helsinki. The anonymized data set generated and analyzed during the current study is available from the corresponding author upon reasonable request.

This multicenter retrospective observational study was conducted between April and September 2025. We retrospectively included eligible adult (>18 years) patients for whom serum samples were available at all three protocol-defined time points, admitted to the hospital with sepsis, positive blood cultures for Gram-negative bacteria, and ARF. Exclusion criteria were malignancy, alcohol or drug abuse, pregnancy, and breastfeeding.

Sepsis was defined as life-threatening organ dysfunction caused by a dysregulated host response to infection (1). To ensure a more homogeneous study population, we also required the presence of ARF as the organ dysfunction criterion. ARF was defined as acute hypoxemia, identified by peripheral oxygen saturation (SpO₂) <90% on room air and/or signs of clinical respiratory distress, including tachypnea (>30 breaths/min), labored breathing, or use of accessory respiratory muscles; when arterial blood gas analysis was available, a PaO₂ <60 mmHg supported the diagnosis (17, 18).

For all participants, blood samples for biomarker assessment were obtained at the time of sepsis diagnosis and before the initiation of antibiotic therapy (T0). Additional samples were collected at 3 days (T1) and 7 days (T2) after diagnosis.

By design, all 82 included patients had samples and measurements available at T0, T1, and T2 for calprotectin, routine biomarkers, cytokines, and growth factors. Accordingly, every biomarker analysis was performed in the complete cohort (*n* = 82; 64 survivors and 18 non-survivors); no patient was excluded from any analysis, and no statistical imputation was performed.

Demographic characteristics, type of respiratory support at sepsis onset, sequential organ failure assessment 2 (SOFA-2) score (19), white blood cell (WBC) count, absolute neutrophil, lymphocyte, and platelet counts, creatinine, bilirubin, and blood culture isolates were extracted from the medical records. The neutrophil-to-lymphocyte ratio (NLR) was calculated as the absolute neutrophil count divided by the absolute lymphocyte count and was considered a composite inflammatory index reflecting the balance between innate immune activation and adaptive immune responses (20). Respiratory support was classified as conventional oxygen therapy (nasal prongs, face mask, or Venturi mask), high-flow nasal cannula, non-invasive ventilation, or, in the most severe cases, invasive mechanical ventilation via orotracheal intubation (17). Finally, the 28-day mortality after sepsis diagnosis was also recorded.

### Biomarker measurement and laboratory procedures

Calprotectin was quantified in serum with a commercially available Calprest NG-S (ECL) ELISA kit (Eurospital Diagnostic, Trieste, Italy). The assay is based on antibody-mediated recognition of calprotectin and colorimetric signal generation: calprotectin contained in diluted serum binds to antibodies coated on the microplate, after which an HRP-labeled detection antibody forms the immunocomplex. Enzymatic conversion of the substrate generates a measurable color signal, whose intensity is directly related to analyte concentration. Final values were extrapolated from the assay standard curve. Blood tubes were centrifuged at 3,000 × *g* for 10 min at room temperature, and serum was separated within 3 h after collection. At least 500 μL of serum was required for testing. When immediate analysis was not feasible, aliquots were stored at −20°C in screw-cap tubes (0.5–2 mL) and exposed to a single freeze-thaw cycle. The analytical range of the method was 0–150 ng/mL; according to the manufacturer, intra-assay and inter-assay imprecision in serum were <7% and <12.2%, respectively (8).

Routine biochemical markers, including C-reactive protein (CRP) and procalcitonin (PCT), were measured by chemiluminescent immunoassay (21). Cytokine profiling was performed on serum samples using a multiplex biochip platform (Cytokine and Growth Factors Array, Randox Laboratories, Crumlin, UK) for IL-1α, IL-1β, IL-2, IL-4, IL-6, IL-8, IL-10, IFN-γ, MCP-1, TNF-α, endothelial growth factor (EGF), and vascular endothelial growth factor (VEGF). In brief, samples were incubated on the antibody-coated biochip at 37°C for 1 h, washed, and then exposed for an additional hour to an HRP-conjugated secondary antibody. Signal acquisition was carried out with the Evidence Investigator analyzer (Randox Laboratories, Crumlin, UK) using the Cytokines Array I High Sensitivity kit, and concentrations were computed with the manufacturer’s software (22). The reportable ranges, corresponding intra-assay and inter-assay coefficients of variation, are reported in Table S1 in the Electronic Supplemental Materials (ESM) (22).

To limit measurement bias, laboratory staff were unaware of all clinical data and outcomes. Sample preparation and analytical procedures followed a predefined standardized workflow, and no patient-level clinical information was disclosed to the operators during any phase of testing.

### Statistics

Data distribution was assessed using the Shapiro-Wilk test. Because biomarker values were right-skewed and logarithmic transformation did not restore normality, non-parametric methods were used. Continuous variables are reported as median [interquartile range], and categorical variables as counts (percentages). Temporal changes across T0, T1, and T2 were assessed using the Friedman test followed, when significant, by Dunn’s pairwise comparisons with Bonferroni correction. Between-group comparisons at each time point were performed using two-sided Mann-Whitney U tests.

Calprotectin was the principal biomarker of interest, whereas analyses of routine biomarkers, cytokines, and growth factors were exploratory. To account for multiple testing, the Benjamini-Hochberg procedure was applied to control the false-discovery rate across the 63 secondary biomarker-by-time comparisons (23). Differences in longitudinal trajectories between survivors and non-survivors were evaluated using repeated-measures mixed-effects models, including group, time, and their interaction, with false-discovery-rate correction across the 22 trajectory tests. Significant pairwise differences were summarized as median differences with 95% confidence intervals.

Exploratory two-sided Spearman analyses assessed relationships between baseline calprotectin and selected measures of disease severity, routine biomarkers, cytokines, and growth factors. Benjamini-Hochberg correction was applied across the 22 correlations, which were not used for predictor selection.

Receiver operating characteristic (ROC) analyses were performed for SOFA-2 and calprotectin and, exploratorily, for secondary biomarkers showing nominal unadjusted between-group differences. AUCs and their 95% confidence intervals were estimated using the DeLong method (24), optimal thresholds were identified using the Youden index, and exact binomial confidence intervals were calculated for sensitivity, specificity, and predictive values. The AUCs of SOFA-2 and baseline calprotectin were compared using the DeLong method.

Given the limited number of deaths, clinically selected parsimonious models were fitted using Firth bias-reduced logistic regression (25). Separate models included age, SOFA-2, and one log^2^-transformed baseline biomarker—calprotectin, NLR, CRP, or PCT. Model discrimination was assessed using the AUC and internally validated using 500 stratified bootstrap resamples to estimate optimism-corrected performance (26). An additional model, including age, SOFA-2, calprotectin, NLR, and CRP, was considered exploratory. No data-driven variable selection or exhaustive search for biomarker combinations was performed.

All tests were two-sided, with *P* < 0.05 considered statistically significant. Complete analytical definitions, multiple-testing procedures, effect-size calculations, diagnostic-accuracy measures, model specifications, and internal-validation methods are provided in the Extended Statistical Analysis Plan in the ESM.

## RESULTS

Eighty-two adult patients with Gram-negative bloodstream infection were included. Sixty-four patients (78%) survived, whereas 18 (22%) died. The characteristics of the overall population, and of survivors and non-survivors separately, are shown in Table 1, including microbiological findings. Survivors and non-survivors were similar with respect to baseline characteristics, department of admission, and infecting bacteria. Non-survivors had a more severe clinical presentation, characterized by higher SOFA-2 scores and greater use of non-invasive or invasive ventilatory support, as compared to survivors (see Table 1). Overall, survivors and non-survivors differed primarily in baseline clinical severity and respiratory-support requirements rather than in demographic characteristics or infecting species.

**TABLE 1:** Characteristics of the population^*b*^

	Overall (*n* = 82)	Survivors (*n* = 64)	Non-survivors (*n* = 18)	*P* value
Male gender—*n* (%)	50 (61.0)	38 (59.4)	12 (66.7)	0.785
Age (years)	72.5 [61.8; 82.3]	71.0 [60.3; 81.8]	75.5 [68.5; 83.3]	0.232
SOFA-2 score	5 [3; 7]	4 [2; 8]	8 [6; 11]	<0.001
Department of admission—*n* (%)				
Wards	53 (64.6)	46 (71.9)	7 (38.9)	0.199
HDU	10 (12.2)	7 (10.9)	3 (16.7)	
ICU	19 (23.2)	11 (17.2)	8 (44.4)	
Respiratory support—*n* (%)				
COT	10 (12.2)	10 (15.6)	0 (0.0)	<0.001
HFNC	32 (39.0)	32 (50.0)	0 (0.0)	
NIV	22 (26.8)	12 (18.8)	10 (55.6)	
iMV	18 (22.0)	10 (15.6)	8 (44.4)	
Blood culture—*n* (%)^*a*^				
*A. baumannii*	3 (3.7)	3 (4.7)	0 (0.0)	0.379
*A. hydrophila*	1 (1.2)	1 (1.6)	0 (0.0)	
*A. pittii*	1 (1.2)	1 (1.6)	0 (0.0)	
*E. coli*	31 (37.8)	27 (42.2)	4 (22.2)	
*E. hormaechei*	2 (2.4)	2 (3.1)	0 (0.0)	
*F. nucleatum*	1 (1.2)	1 (1.6)	0 (0.0)	
*K. pneumoniae*	31 (37.8)	21 (32.8)	10 (55.6)	
*P. aeruginosa*	5 (6.1)	3 (4.7)	2 (11.1)	
*P. mirabilis*	7 (8.5)	4 (6.3)	3 (16.7)	
*P. stutzeri*	1 (1.2)	1 (1.6)	0 (0.0)	
*Proteus* spp.	1 (1.2)	1 (1.6)	0 (0.0)	
*S. marcescens*	1 (1.2)	0 (0.0)	1 (5.6)	

^*a*^ The percentages exceed 100% because multiple isolates were recovered from some patients.

^*b*^ SOFA-2, sequential organ failure assessment 2; HDU, high-dependency unit; ICU, intensive care unit; COT, conventional oxygen therapy; HFNC, high flow nasal cannula; NIV, non-invasive ventilation; iMV, invasive mechanical ventilation.

Complete numerical results, including all pairwise comparisons, effect sizes, unadjusted *P* values, false-discovery-rate-adjusted q values, formal comparisons of longitudinal trajectories between outcome groups, and ROC parameters, are provided in Tables S2 through S6 in the ESM.

### Calprotectin

**Fig 1:**
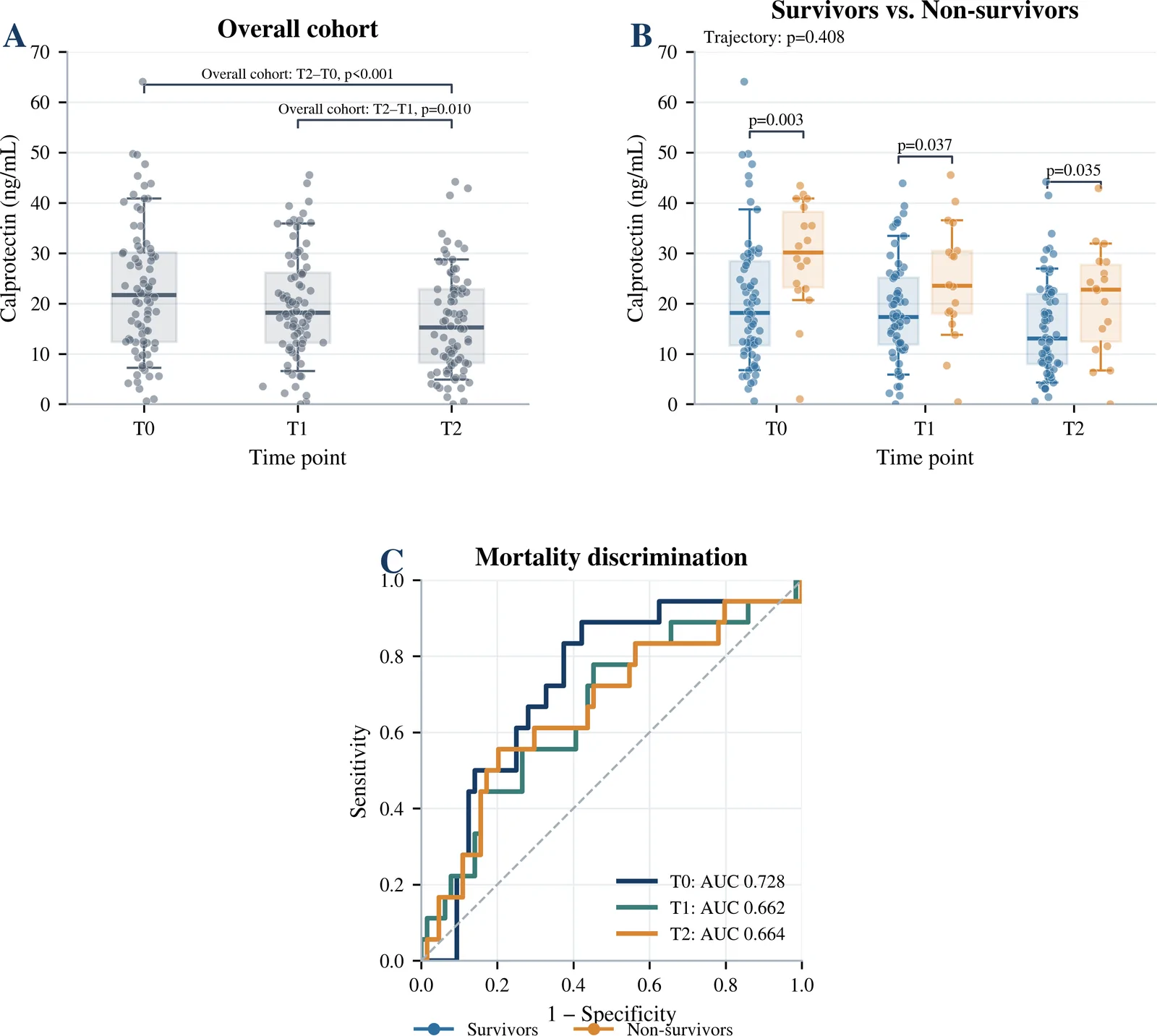
Temporal profile and mortality discrimination of serum calprotectin. (**A**) Serum calprotectin concentrations in the overall cohort at sepsis diagnosis before antibiotic therapy (T0), day 3 (T1), and day 7 (T2). (**B**) Serum calprotectin concentrations in survivors and non-survivors across the three time points. The trajectory *P* value is from the formal group-by-time test and evaluates whether the longitudinal pattern differed between outcome groups. Horizontal brackets identify significant pairwise changes in the overall cohort or unadjusted survivor-versus-non-survivor differences at the specified time point. (**C**) Receiver operating characteristic curves for discrimination of 28-day mortality by calprotectin at T0, T1, and T2; the corresponding AUCs were 0.728, 0.662, and 0.664, respectively. All panels include the complete cohort of 82 patients (64 survivors and 18 non-survivors). Box plots show all patient-level observations, the median and interquartile range, with whiskers extending from the 10th to the 90th percentile.

The principal findings for calprotectin are summarized in Fig. 1. In the overall cohort, calprotectin concentrations changed significantly over time and were lower at T2 than at both T0 (*P* < 0.001) and T1 (*P* = 0.010) (Table S2 in the ESM).

Significant temporal changes were also observed within both outcome groups. Among survivors, calprotectin concentrations were lower at T2 than at T0 (*P* < 0.001) and T1 (*P* = 0.040). Among non-survivors, concentrations were lower at T2 than at T0 (*P* < 0.001) (Table S3 in the ESM). Nevertheless, calprotectin remained significantly higher in non-survivors than in survivors at T0 (*P* = 0.003), T1 (*P* = 0.037), and T2 (*P* = 0.035) (Table S4 in the ESM). The longitudinal trajectory of calprotectin did not differ between survivors and non-survivors (*P* = 0.408; Table S5 in the ESM).

Because calprotectin differed between outcome groups at all three time points, its ability to discriminate 28-day mortality was evaluated using ROC analysis. Calprotectin showed moderate discrimination at T0, T1, and T2, with the highest AUC observed at T0 and slightly lower performance at T1 and T2 (Table S6 in the ESM).

### Biomarkers

In the overall cohort, WBC count, NLR, CRP, and PCT changed significantly over time, with values at T2 being lower than those observed at both T0 and T1 (Table S2 in the ESM). Similar reductions were observed among survivors, in whom WBC count, NLR, CRP, and PCT were significantly lower at T2 than at both previous time points. Among non-survivors, WBC count and CRP did not change significantly over time, whereas NLR was lower at T2 than at T1 (*P* = 0.037) and PCT was lower at T2 than at T0 (*P* = 0.011) (Table S3 in the ESM). Between-group comparisons showed no significant differences in WBC count or PCT at any time point. Conversely, at T2, NLR and CRP were nominally higher in non-survivors than in survivors (*P* = 0.002 and *P* = 0.013, respectively) (Table S4 in the ESM).

**Fig 2:**
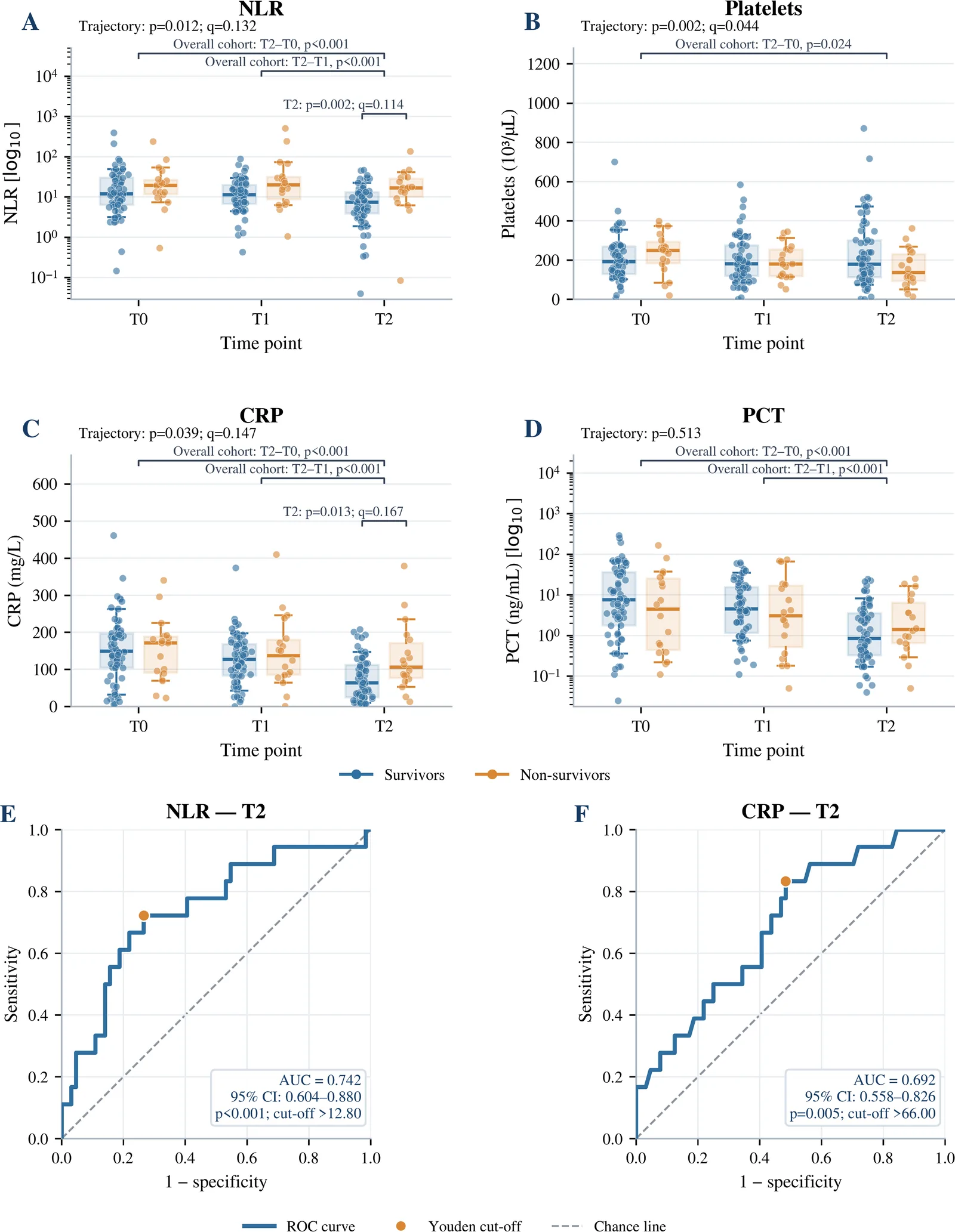
Longitudinal profiles of selected routine biomarkers according to 28-day outcome. Longitudinal distributions of (**A**) NLR, (**B**) platelet count, (**C**) CRP, and (**D**) PCT at sepsis diagnosis before antibiotic therapy (T0), day 3 (T1), and day 7 (T2) in survivors and non-survivors. Trajectory *P* values are from the formal group-by-time tests; q values were obtained by Benjamini-Hochberg false-discovery-rate correction across the 22 trajectory tests. Horizontal brackets identify significant pairwise temporal changes in the overall cohort or nominal unadjusted survivor-versus-non-survivor differences at the specified time point. For time point-specific between-group comparisons, q values were obtained by false-discovery-rate correction across the 63 secondary biomarker-by-time comparisons. Only the platelet trajectory remained statistically significant after correction across the trajectory tests. (**E**) ROC curve for NLR at T2 and (**F**) ROC curve for CRP at T2 for discrimination of 28-day mortality. The corresponding AUCs were 0.742 (95% CI, 0.604–0.880) for NLR and 0.692 (95% CI, 0.558–0.826) for CRP. The indicated points correspond to the Youden-derived cut-offs (>12.80 for NLR and >66.00 mg/L for CRP). These ROC analyses were exploratory because the corresponding between-group comparisons did not remain statistically significant after false-discovery-rate correction. Box plots show every patient-level observation, the median and interquartile range, with whiskers extending from the 10th to the 90th percentile. NLR and PCT are displayed on logarithmic y-axes for visualization only; original values were plotted, and no observations were transformed or excluded. AUC, area under the receiver operating characteristic curve; CI, confidence interval; CRP, C-reactive protein; NLR, neutrophil-to-lymphocyte ratio; PCT, procalcitonin; ROC, receiver operating characteristic.

ROC analysis at T2 showed moderate discriminative performance for NLR, with an AUC of 0.742 (95% CI, 0.604–0.880), and for CRP, with an AUC of 0.692 (95% CI, 0.558–0.826) (Fig. 2; Fig. S4 and Table S6 in the ESM).

Regarding the remaining routine biomarkers, neutrophil counts declined significantly over time in the overall cohort and among survivors, but not among non-survivors, and were nominally higher in non-survivors at T1 and T2. Lymphocyte counts increased from T1 to T2 in the overall cohort and were nominally lower in non-survivors at T2. Platelet counts decreased mainly among non-survivors, whereas creatinine and bilirubin showed modest temporal reductions, predominantly among survivors. Nominal differences in longitudinal trajectories were observed for WBC count, neutrophil count, NLR, platelet count, bilirubin, and CRP. After false-discovery-rate correction across the 22 formal trajectory comparisons, only the platelet trajectory remained statistically different between outcome groups (q = 0.044; Table S5 in the ESM). None of the time point-specific comparisons involving secondary routine biomarkers remained statistically significant after false-discovery-rate correction. Complete numerical results are reported in Tables S2 through S6, and the corresponding longitudinal profiles are presented in Fig. 2; Fig. S1 (in the ESM).

### Cytokines

**Fig 3:**
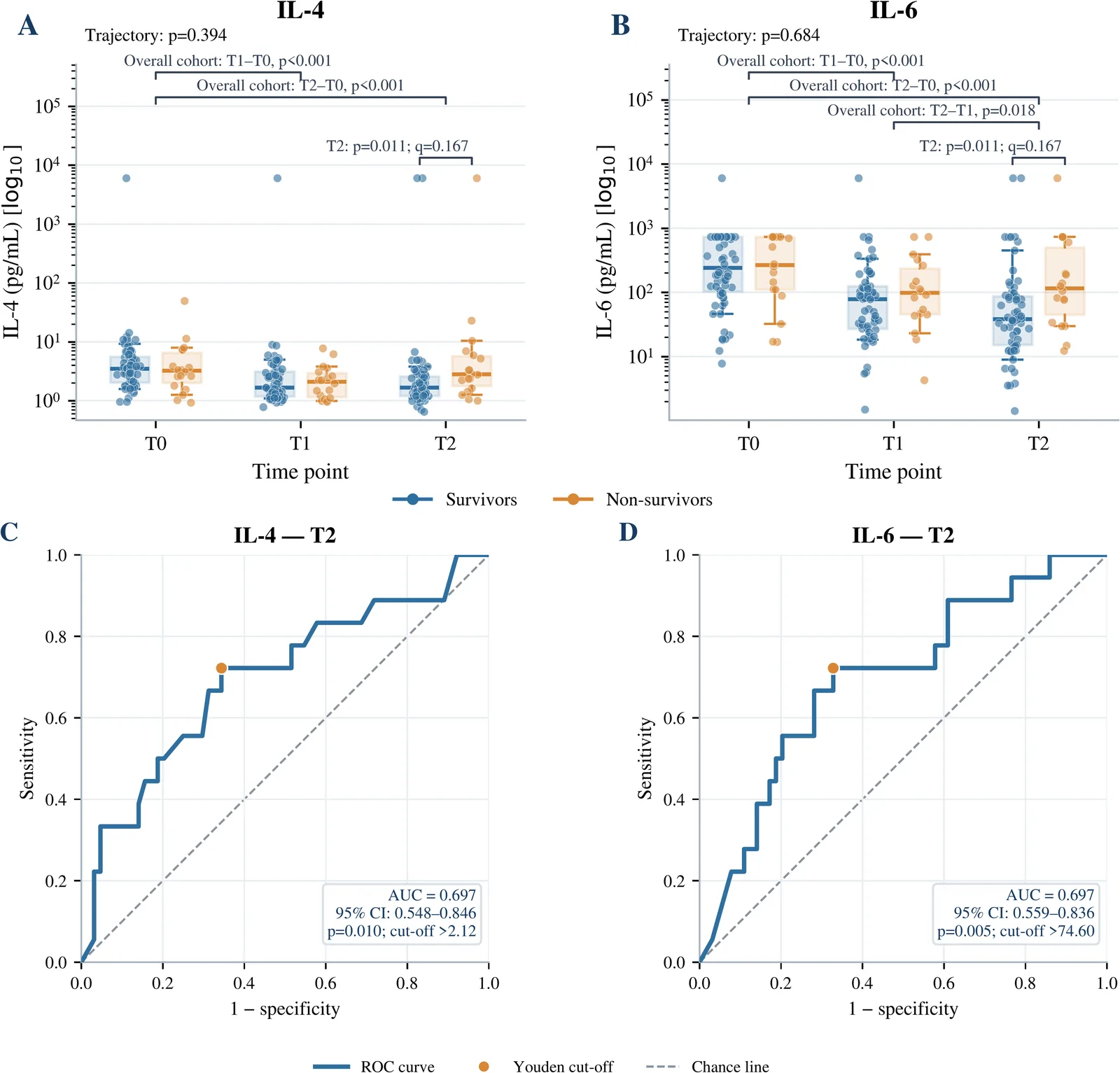
Longitudinal profiles of selected cytokines according to 28-day outcome. Longitudinal distributions of (**A**) interleukin (IL)-4 and (**B**) IL-6 at sepsis diagnosis before antibiotic therapy (T0), day 3 (T1), and day 7 (T2) in survivors and non-survivors. Trajectory *P* values are from the formal group-by-time tests. Horizontal brackets identify significant pairwise temporal changes in the overall cohort or nominal unadjusted survivor-versus-non-survivor differences at the specified time point. For time point-specific between-group comparisons, q values were obtained by Benjamini–Hochberg false-discovery-rate correction across the 63 secondary biomarker-by-time comparisons. At T2, IL-4 and IL-6 were nominally higher in non-survivors, but neither comparison remained statistically significant after false-discovery-rate correction. (**C**) ROC curve for IL-4 at T2 and (**D**) ROC curve for IL-6 at T2 for discrimination of 28-day mortality. The corresponding AUCs were 0.697 (95% CI, 0.548–0.846) for IL-4 and 0.697 (95% CI, 0.559–0.836) for IL-6. The indicated points correspond to the Youden-derived cut-offs (>2.12 pg/mL for IL-4 and >74.60 pg/mL for IL-6). These ROC analyses were exploratory because the corresponding between-group comparisons did not remain statistically significant after false-discovery-rate correction. Box plots show every patient-level observation, the median and interquartile range, with whiskers extending from the 10th to the 90th percentile. Cytokine concentrations are displayed on logarithmic y-axes for visualization only; original values were plotted and no observations were transformed or excluded. AUC, area under the receiver operating characteristic curve; CI, confidence interval; IL, interleukin; ROC, receiver operating characteristic.

The principal findings for IL-4, IL-6, IL-10, MCP-1, and TNF-α are summarized in Fig. 3; Fig. S2 (in the ESM).

In the overall cohort, IL-4, IL-6, IL-10, MCP-1, and TNF-α changed significantly over time. IL-6 declined across all three pairwise comparisons, whereas IL-4, IL-10, MCP-1, and TNF-α were mainly lower at T1 and/or T2 than at T0 (Table S2 in the ESM). Among survivors, IL-4 and IL-6 decreased from T0, with an additional T1-to-T2 reduction observed only for IL-6. IL-10, MCP-1, and TNF-α were lower at T1 and T2 than at T0. Among non-survivors, overall temporal changes were observed for IL-6, IL-10, and TNF-α, although corrected pairwise differences were limited to IL-6 at T2 versus T0 and IL-10 at T1 and T2 versus T0 (Table S3 in the ESM).

No cytokine differed between survivors and non-survivors at T0 or T1. At T2, non-survivors had nominally higher IL-4 (*P* = 0.011) and IL-6 (*P* = 0.011). Differences in MCP-1 (*P* = 0.055), TNF-α (*P* = 0.064), and IL-10 (*P* = 0.076) did not reach statistical significance. Neither IL-4 nor IL-6 remained significant after false-discovery-rate correction (both q = 0.167), and no cytokine showed a longitudinal trajectory that differed between outcome groups (Tables S4 and S5 in the ESM).

In exploratory ROC analyses at T2, the AUC was 0.697 for IL-4 (95% CI, 0.548–0.846) and 0.697 for IL-6 (95% CI, 0.559–0.836) (Fig. 3; Fig. S5 and Table S6 in the ESM). Because the corresponding between-group comparisons did not remain significant after false-discovery-rate correction, these estimates should be interpreted as descriptive and hypothesis-generating.

Regarding the remaining cytokines, IL-1β, IL-2, and IFN-γ showed modest temporal reductions in the overall cohort and among survivors, whereas IL-1α and IL-8 did not show significant temporal changes. None of these cytokines differed between survivors and non-survivors at any time point, and none showed a longitudinal trajectory that differed between outcome groups. Complete results are reported in Tables S2 through S5 in the ESM, and the corresponding profiles are presented in Fig. S2.

### Growth factors

Growth factors provided limited outcome-related information. Neither EGF nor VEGF changed significantly over time in the overall cohort or among survivors. Among non-survivors, EGF showed an overall temporal change and was lower at T2 than at T0, whereas VEGF did not vary significantly. However, neither growth factor differed between survivors and non-survivors at any time point, and neither showed a longitudinal trajectory that differed between outcome groups. The isolated within-group EGF change was therefore not interpreted as evidence of an outcome-specific trajectory (Tables S2 through S5 in the ESM). The corresponding profiles are presented in Fig. S3.

### Relationships between baseline calprotectin and other clinical and biological measures

Exploratory baseline analyses showed that calprotectin correlated positively with WBC count, neutrophil count, NLR, CRP, and VEGF. The weaker correlation with SOFA-2 was nominally significant before correction but did not remain significant after false-discovery-rate adjustment. No FDR-significant correlations were observed with PCT, lymphocyte or platelet counts, creatinine, bilirubin, the measured cytokines, or EGF. These findings indicate partial overlap between calprotectin and neutrophil-related or conventional inflammatory measures, without evidence of a strong relationship with any single comparator (detailed data are reported in Table S7 and summarized graphically in Fig. S6 in the ESM).

### Prognostic performance of SOFA-2 and multivariable models

SOFA-2 alone showed strong discrimination for 28-day mortality, with an AUC of 0.897 (95% CI, 0.828–0.965; *P* < 0.001). The optimal threshold in this cohort was a SOFA-2 score ≥5, which yielded a sensitivity of 100.0%, a specificity of 60.9%, a positive predictive value of 41.9%, and a negative predictive value of 100.0%. SOFA-2 showed significantly greater discrimination than calprotectin at T0 (AUC, 0.897 versus 0.728; difference in AUC, 0.168; *P* = 0.027).

The clinical model, including age and SOFA-2, had an apparent AUC of 0.899 (95% CI, 0.821–0.978) and an optimism-corrected AUC of 0.893. In this model, both age (OR, 1.81 per 10-year increase; 95% CI, 1.11–3.46; *P* = 0.015) and SOFA-2 (OR, 1.98 per one-point increase; 95% CI, 1.49–2.90; *P* < 0.001) were independently associated with mortality (Table 2).

**TABLE 2:** Multivariable prognostic models for 28-day mortality^*a*^

Model	Biomarker-adjusted OR (95% CI)	*P* value	Apparent AUC (95% CI)	Optimism-corrected AUC	ΔAUC versus age + SOFA-2	DeLong *P*
Age + SOFA-2	–^*b*^	–	0.899 (0.821–0.978)	0.893	Reference	–
Age + SOFA-2 + calprotectin	2.07 per doubling (1.14–4.20)	0.018	0.922 (0.854–0.990)	0.907	+0.023	0.117
Age + SOFA-2 + NLR	1.05 per doubling (0.66–1.61)	0.838	0.897 (0.817–0.977)	0.886	−0.003	0.402
Age + SOFA-2 + CRP	1.08 per doubling (0.57–2.34)	0.823	0.904 (0.827–0.980)	0.889	+0.004	0.382
Age + SOFA-2 + PCT	0.91 per doubling (0.72–1.14)	0.411	0.901 (0.824–0.978)	0.886	+0.002	0.851

^*a*^ Odds Ratios (ORs) and 95% confidence intervals (95% CI) were estimated using Firth’s bias-reduced penalized logistic regression. All models included age and SOFA-2. Age was expressed per 10-year increase, SOFA-2 per one-point increase, and biomarkers per doubling of the T0 value. Optimism-corrected AUCs were estimated using 500 stratified bootstrap resamples. Differences between correlated AUCs were evaluated using the DeLong method. AUC, area under the receiver operating characteristic curve; CRP, C-reactive protein; NLR, neutrophil-to-lymphocyte ratio; PCT, procalcitonin.

^*b*^ "–" mean not applicable.

After adjustment for age and SOFA-2, baseline calprotectin remained independently associated with 28-day mortality (OR, 2.07 per doubling; 95% CI, 1.14–4.20; *P* = 0.018). The model, including age, SOFA-2, and calprotectin, had an apparent AUC of 0.922 (95% CI, 0.854–0.990) and an optimism-corrected AUC of 0.907. However, the increase in apparent AUC relative to the age-plus-SOFA-2 model was modest and did not reach statistical significance (ΔAUC, 0.023; *P* = 0.117) (Table 2).

Baseline NLR, CRP, and PCT were not independently associated with mortality after adjustment for age and SOFA-2 and did not materially improve model discrimination. The exploratory model combining calprotectin, NLR, and CRP with age and SOFA-2 had an apparent AUC of 0.920 and an optimism-corrected AUC of 0.898, providing no clear advantage over the simpler model containing age, SOFA-2, and calprotectin.

**Fig 4:**
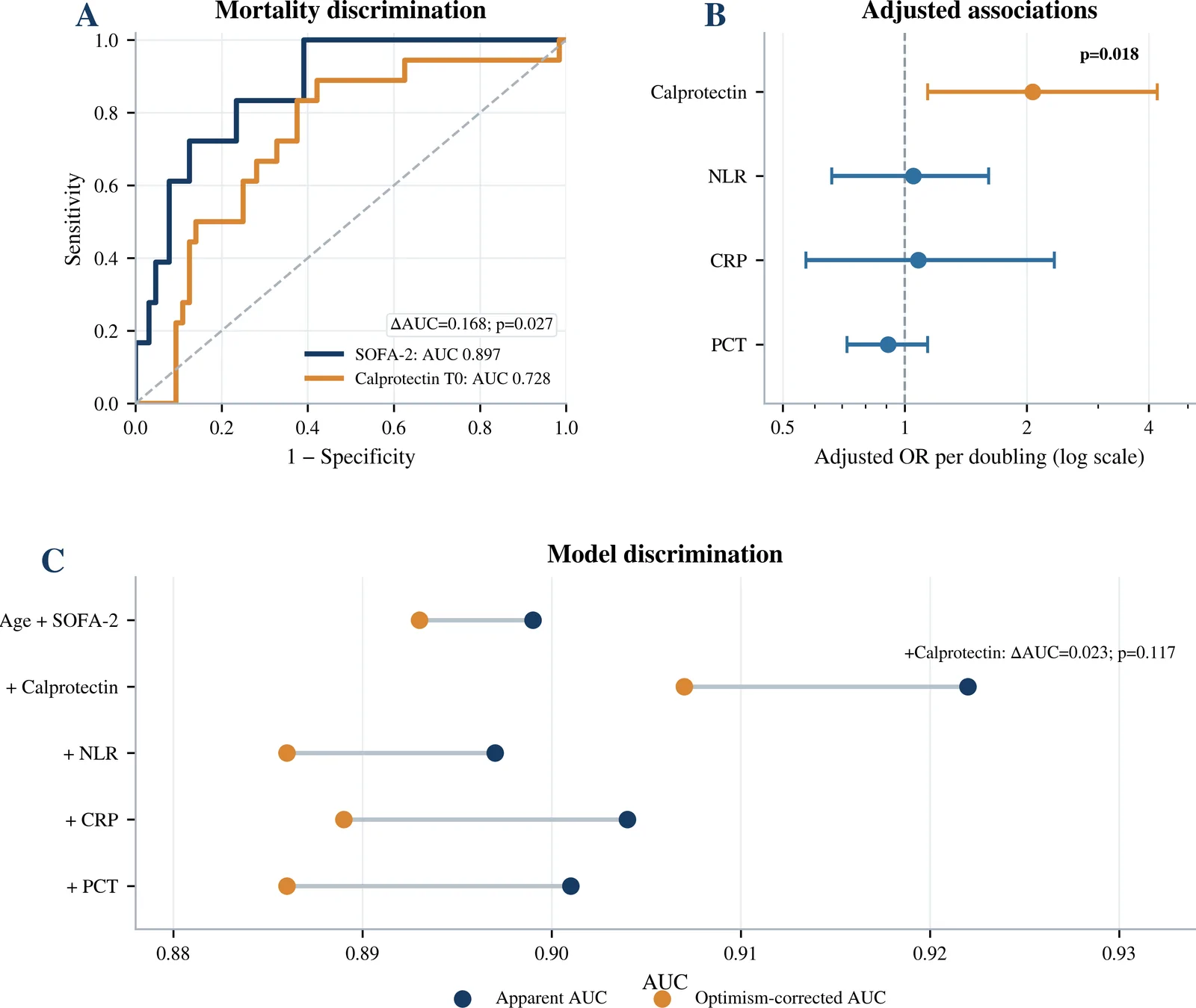
Prognostic discrimination and adjusted associations with 28-day mortality. (**A**) Receiver operating characteristic curves comparing SOFA-2 with baseline calprotectin for discrimination of 28-day mortality. AUCs were 0.897 for SOFA-2 and 0.728 for calprotectin at T0; the difference was evaluated using the DeLong test for correlated curves. (**B**) Adjusted odds ratios and 95% confidence intervals for each baseline biomarker from separate Firth bias-reduced penalized logistic regression models that included age, SOFA-2, and one biomarker at T0. Biomarkers were log2-transformed; odds ratios therefore represent the change in the odds of mortality associated with a doubling of the biomarker concentration. (**C**) Apparent and bootstrap optimism-corrected AUCs for the clinical model and models formed by adding one baseline biomarker. The displayed delta AUC and *P* value refer to the exploratory comparison between the age-plus-SOFA-2 model and the model additionally containing calprotectin. Models were clinically selected and parsimonious; no data-driven variable selection procedure was used. Model comparisons are exploratory. AUC, area under the receiver operating characteristic curve; CI, confidence interval; CRP, C-reactive protein; NLR, neutrophil-to-lymphocyte ratio; OR, odds ratio; PCT, procalcitonin; SOFA-2, sequential organ failure assessment 2.

Overall, SOFA-2 was the dominant prognostic discriminator, whereas baseline calprotectin retained an independent association with mortality but provided only a modest and statistically non-significant improvement in model discrimination (Fig. 4).

## DISCUSSION

**Fig 5:**
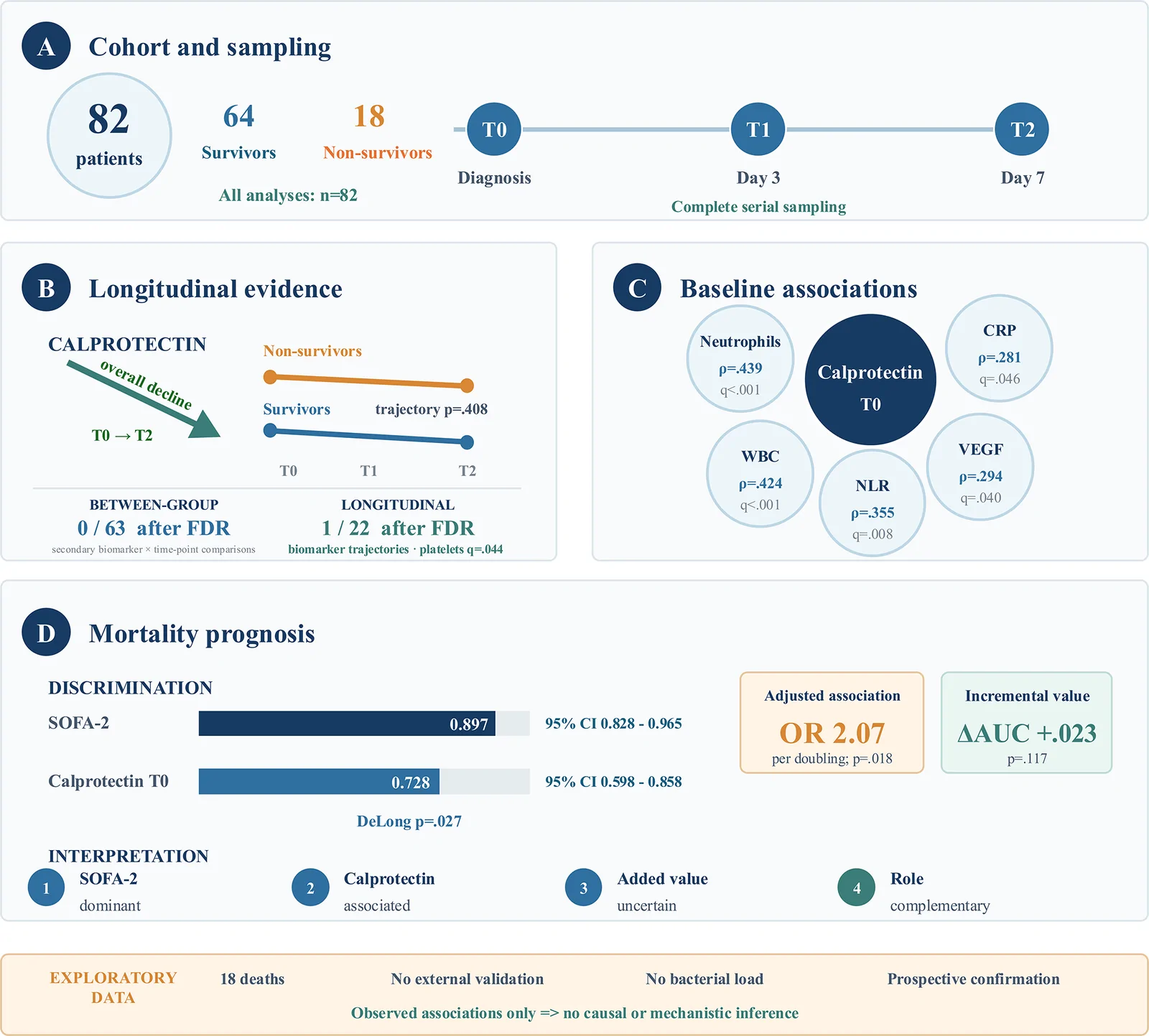
Integrated descriptive summary of the study design, longitudinal findings, baseline associations, and prognostic analyses. (**A**) Study cohort (*n* = 82; 64 survivors and 18 non-survivors) and protocol-defined serum sampling at sepsis diagnosis before antibiotic administration (T0), day 3 (T1), and day 7 (T2). All analyses included the complete cohort of 82 patients. (**B**) Summary of the longitudinal findings. Calprotectin declined over the first week, with no evidence of a different longitudinal trajectory between survivors and non-survivors (*P* = 0.408). After FDR correction, none of the 63 secondary biomarker-by-time between-group comparisons remained statistically significant, whereas only 1 of the 22 longitudinal trajectory tests remained significant (platelets, q = 0.044). (**C**) Exploratory baseline Spearman correlations between calprotectin and clinical or biological measures that remained significant after FDR correction. (**D**) Mortality discrimination and adjusted association analyses, including the relative discrimination of SOFA-2 and baseline calprotectin, the adjusted association of calprotectin with 28-day mortality, and its incremental contribution beyond the clinical model. The lower panel summarizes the exploratory nature of the findings and key study limitations. The figure provides a descriptive synthesis of observed statistical associations and does not imply distinct outcome-specific immune trajectories, cellular mechanisms, or causal pathways. Secondary biomarker analyses, correlation analyses, and model comparisons should be considered exploratory and hypothesis-generating. AUC, area under the receiver operating characteristic curve; CI, confidence interval; CRP, C-reactive protein; FDR, false-discovery rate; NLR, neutrophil-to-lymphocyte ratio; OR, odds ratio; SOFA-2, sequential organ failure assessment 2; VEGF, vascular endothelial growth factor; WBC, white blood cell count.

In this multicenter cohort of adults with sepsis due to Gram-negative bloodstream infection and concomitant ARF, calprotectin declined during the first week and was higher in non-survivors at each time point, but its longitudinal trajectory did not differ between outcome groups. None of the secondary routine or immune biomarker comparisons remained statistically significant after false-discovery-rate correction. SOFA-2 was the strongest early discriminator of mortality and outperformed baseline calprotectin. Although baseline calprotectin remained associated with mortality after adjustment for age and SOFA-2, its addition produced only a modest and statistically non-significant improvement in discrimination. These findings support interpreting calprotectin as a potentially complementary biomarker rather than a standalone prognostic tool. An integrated summary of the study design and principal findings is provided in Fig. 5.

A principal focus of this study was the longitudinal evaluation of calprotectin in a clinically defined cohort with documented Gram-negative bacteremia. Calprotectin is a neutrophil-derived S100A8/A9 complex released during myeloid activation and inflammatory tissue injury (9, 10, 27). Previous clinical studies have mainly examined its diagnostic role: a meta-analysis supported its potential usefulness for early sepsis diagnosis (12), Larsson et al. reported greater diagnostic performance than PCT in critically ill patients (28), and the prospective CASCADE trial found an association between circulating calprotectin, bacterial infection, and sepsis severity (11).

Our findings can be compared with the limited and heterogeneous literature on serial calprotectin measurements. Huang et al. reported dynamic changes in serum calprotectin in postoperative ICU patients and investigated their potential diagnostic relevance (16). Wirtz et al. found that high admission calprotectin predicted adverse outcomes in critically ill septic patients and that increasing concentrations between day 1 and day 7 were associated with survival (13). By contrast, de Guadiana-Romualdo et al. reported that baseline and 24-h calprotectin alone lacked sufficient prognostic accuracy for 28-day mortality, although serial combined-marker models improved performance (15). In our cohort, the higher absolute calprotectin concentrations in non-survivors were not accompanied by a different longitudinal trajectory, indicating a shared temporal pattern across outcome groups. The observed decline should therefore not be interpreted as a specific marker of treatment response or resolution of systemic inflammation.

The microbiological restriction of the cohort is relevant to interpretation. Gram-negative bacteremia may involve systemic exposure to lipopolysaccharide and other pathogen-associated molecular patterns capable of activating innate immune pathways, including TLR4 signaling (29–31), and experimental studies have implicated S100A8/A9 in sepsis-related inflammatory injury (27, 32). However, endotoxin concentrations, bacterial load, neutrophil activation, and pulmonary tissue responses were not measured. The observed calprotectin differences therefore cannot be attributed to greater endotoxin exposure, pathogen burden, or neutrophil-mediated lung injury. Moreover, non-survivors had higher SOFA-2 scores and greater respiratory-support requirements, indicating greater baseline clinical severity. Thus, the microbiological selection provides a defined clinical context, but the underlying biological mechanisms remain uncertain.

Quantification of bacterial load could provide a pathogen-centered measure complementary to host-response biomarkers. Previous studies have associated higher circulating bacterial DNA concentrations with greater disease severity, septic shock, mechanical ventilation, or mortality, although these associations appear to depend on the pathogen and analytical method used (33, 34). Serial measurements may be particularly informative: in a small prospective cohort of Gram-negative bloodstream infections, pathogen DNA load decreased during treatment in survivors but remained stable or increased in non-survivors (35). Moreover, prolonged detection of microbial cell-free DNA has been associated with metastatic infection in patients with *Staphylococcus aureus* or Gram-negative bacteremia (36). These findings support evaluating bacterial load alongside host-response biomarkers in future sepsis studies, particularly as a potential indicator of microbiological clearance and treatment response. However, differences in pathogen growth, sampling time, prior antimicrobial exposure, assay characteristics, and the persistence of DNA from non-viable organisms currently limit its interpretation as a standardized clinical metric. Because bacterial load was not measured in our cohort, its relationship with calprotectin concentrations and clinical outcome could not be assessed.

The longitudinal patterns of PCT and CRP provide additional context for the interpretation of calprotectin. Both markers declined during the first week, particularly among survivors, consistent with previous studies reporting temporal changes in inflammatory biomarkers during sepsis and septic shock (5, 37, 38). However, PCT did not differ between survivors and non-survivors at any time point and showed no evidence of a different longitudinal trajectory according to outcome. CRP showed a later nominal between-group difference at T2 and a nominal difference in longitudinal trajectory, but these secondary findings did not remain significant after false-discovery-rate correction and should therefore be interpreted as exploratory. Differences from previous studies reporting prognostic associations for PCT or CRP kinetics may reflect variation in study populations, sampling schedules, infection characteristics, and illness severity (37–39).

The exploratory correlation analysis provided additional context for the interpretation of baseline calprotectin. Calprotectin showed moderate positive correlations with neutrophil and WBC counts and weaker correlations with NLR, CRP, and VEGF after false-discovery-rate correction. The associations with neutrophil-related measures are consistent with the abundance of S100A8/A9 in neutrophils and its involvement in inflammatory signaling (9, 10). An association between circulating calprotectin and CRP has also been reported in critically ill and septic patients (13). These correlations indicate partial biomarker overlap but do not identify the cellular source of circulating calprotectin or establish shared biological pathways.

Among cellular biomarkers, NLR showed the highest exploratory individual-biomarker AUC at T2, consistent with previous evidence associating elevated NLR with sepsis severity and mortality (40).

Survivors showed reductions in neutrophil count and NLR together with an increase in lymphocyte count, whereas non-survivors had nominally higher NLR and lower lymphocyte counts at T2. These secondary comparisons did not remain significant after false-discovery-rate correction. Although abnormalities in myelopoiesis and persistent lymphopenia have been associated with adverse outcomes in sepsis (41–43), NLR is an indirect composite measure and the present findings should not be interpreted as evidence of a specific immune phenotype.

Cytokine findings were exploratory. Non-survivors had nominally higher IL-4 and IL-6 levels at T2, whereas no between-group differences were observed at T0 or T1. Neither comparison remained statistically significant after false-discovery-rate correction, and no cytokine showed a longitudinal trajectory that differed between outcome groups. This pattern is consistent with the heterogeneous and pleiotropic cytokine response described in sepsis and with the limited reproducibility of cytokine-based prognostic stratification (44, 45). Given the multiplicity of comparisons, the limited number of deaths, and the absence of robust trajectory differences, these findings should be considered hypothesis-generating and do not identify a reproducible prognostic cytokine signature, a distinct immune phenotype, or a causal pathway.

Growth factors provided limited outcome-related information. Neither EGF nor VEGF differed significantly between survivors and non-survivors at any time point. Previous studies have reported heterogeneous prognostic associations for growth factors in sepsis, including a possible favorable association for EGF and an association between elevated VEGF and adverse outcomes (46, 47). In the present cohort, however, the findings do not support conclusions regarding impaired tissue-repair capacity, and the growth-factor results should therefore be considered exploratory.

The adjusted analyses refine the interpretation of the univariable findings. SOFA-2 alone showed strong discrimination and performed significantly better than baseline calprotectin, emphasizing the prognostic relevance of contemporary clinical organ-dysfunction assessment (19). Nevertheless, calprotectin remained independently associated with mortality after adjustment for age and SOFA-2, with approximately twofold higher odds of death for each doubling of its baseline concentration. However, the addition of calprotectin produced only a small increase in model discrimination, and the difference in AUC was not statistically significant.

By contrast, baseline NLR, CRP, and PCT did not independently improve prediction after adjustment, despite the later outcome-related differences observed for NLR and CRP. Baseline calprotectin was already associated with outcome, whereas serial NLR and CRP measurements described later changes in the inflammatory profile; these observational patterns should not be interpreted as evidence that any marker directly captures treatment response (48, 49). The exploratory multimarker model did not improve optimism-corrected discrimination beyond the simpler model containing age, SOFA-2, and calprotectin. Because only 18 deaths occurred, this finding should not be interpreted as definitive evidence against multimarker approaches; rather, it indicates that increasing model complexity did not provide an evident benefit in the present cohort. Biomarker combinations may be useful in sepsis, but their performance must be evaluated using adequately sized cohorts and appropriate validation procedures (50). External validation in a larger prospective population is required before these models can be considered for clinical application (51).

From a translational perspective, these findings support further evaluation of calprotectin as a complementary component of multimodal risk assessment rather than as a standalone prognostic test (50). Although baseline calprotectin remained independently associated with mortality after adjustment for age and SOFA-2, the improvement in discrimination after its addition to the clinical model was modest and not statistically significant. Therefore, the present results do not support immediate clinical implementation or the use of calprotectin to guide diagnostic or therapeutic decisions. Prospective external validation is required to determine whether calprotectin provides clinically meaningful information when added to established severity scores and routinely available biomarkers (51).

This study has several strengths. The cohort was clinically and microbiologically well defined, being restricted to patients with Gram-negative bloodstream infection and ARF, thereby reducing biological noise relative to generic sepsis cohorts. The availability of three predefined sampling times allowed us to distinguish between absolute biomarker differences and true trajectory differences. The simultaneous assessment of routine biomarkers, calprotectin, cytokines, and growth factors placed calprotectin in a broader immunobiological context. However, the requirement for complete serum sampling at T0, T1, and T2 may have introduced selection bias. In particular, patients who died before the day-7 assessment could not be included in the longitudinal cohort, potentially leading to underrepresentation of the most rapidly fatal clinical courses. The reported associations therefore apply to patients who remained alive and available for sampling through T2 and may not be generalizable to patients who die during the first week after sepsis diagnosis. This potential bias relates to the initial assembly of the longitudinal cohort; once the cohort had been defined, all analyses included all 82 patients.

Several limitations must be acknowledged. The retrospective design precludes causal inference, and the relatively small number of non-survivors may have limited statistical power for formal comparisons of longitudinal trajectories, particularly for cytokines and growth factors. However, the 28-day mortality observed in our cohort was in line with previous studies, providing contextual comparability with previous cohorts (52–54). Sampling was limited to three time points, and no measurements of endotoxin or bacterial load, neutrophil phenotype, NETosis, monocyte tolerance, or tissue-level immune responses were available. The limited sample size also precluded an extensive investigation of correlations across the complete biomarker panel and a data-driven evaluation of multiple biomarker combinations. These relationships should be examined in larger cohorts using prespecified analytical frameworks and independent validation. The limited number of deaths (*n* = 18) restricted model complexity and increased the risk of overfitting. We therefore used parsimonious Firth bias-reduced logistic-regression models with clinically selected predictors, avoided data-driven variable selection, and performed bootstrap internal validation (25, 26). Nevertheless, internal validation cannot replace evaluation in an independent population; therefore, model performance, the optimal SOFA-2 threshold, and the incremental prognostic value of calprotectin should be regarded as cohort-specific until prospectively validated (51). In addition, the large number of secondary biomarker comparisons increased the risk of false-positive findings. None of the 63 secondary biomarker-by-time comparisons remained statistically significant after Benjamini-Hochberg false-discovery-rate correction (23). Accordingly, findings involving routine biomarkers, cytokines, and growth factors should be considered exploratory and require confirmation in larger independent cohorts. Finally, because the cohort was intentionally restricted to Gram-negative bacteraemic sepsis with ARF, our findings may not be generalizable to other sepsis phenotypes, including Gram-positive, fungal, abdominal, or culture-negative sepsis.

### Conclusions

In adults with Gram-negative bloodstream infection, sepsis, and ARF, SOFA-2 was the strongest early discriminator of 28-day mortality. Baseline calprotectin remained independently associated with mortality after adjustment for age and SOFA-2, although its incremental contribution to model discrimination was modest and not statistically significant. Findings involving secondary routine biomarkers, cytokines, and growth factors did not remain significant after false-discovery-rate correction and should be considered exploratory. These results support further evaluation of calprotectin as a complementary component of clinical severity assessment rather than as a standalone prognostic tool.

## Data Availability

The authors will share all the individual participant data collected after de-identification, to researchers who provide a methodologically sound proposal. The full protocol and raw data are available at the corresponding author’s e-mail address.

## SUPPLEMENTAL MATERIAL

Supplemental materials spectrum.01767-26-s0001.docx Supplemental statistical analysis plan, extended results, Tables S1–S7, and Figures S1 to S6.

ASM does not own the copyrights to Supplemental Material that may be linked to, or accessed through, an article. The authors have granted ASM a non-exclusive, world-wide license to publish the Supplemental Material files. Please contact the corresponding author directly for reuse.
