# Supplementary material for: Longitudinal and adjusted prognostic assessment of calprotectin in Gram-negative sepsis with acute respiratory failure
Source: Microbiol Spectr. 2026 Sep 15;14(10):e01767-26. doi: 10.1128/spectrum.01767-26 (PMC13689393; doi:10.1128/spectrum.01767-26)
Supplement: Supplementary file 1 [file spectrum.01767-26-s0001.docx]

*ELECTRONIC SUPPLEMENTARY MATERIALS*

**LONGITUDINAL AND ADJUSTED PROGNOSTIC ASSESSMENT OF CALPROTECTIN IN GRAM-NEGATIVE SEPSIS WITH ACUTE RESPIRATORY FAILURE**

Simona Gigliotti^1^; Michele Manno^1^; Manuela Colosimo^2^; Francesca Divenuto^1^; Francesco Dragone^3^; Marta Greco^3^; Cinzia Peronace^4^; Luigia Gallo^4^; Francesca Trimboli^4^; Pasquale Minchella^2^; Francesca Greco^5^; Alba Silipo^6^; Nadia Marascio^1^; Eugenio Garofalo, MD^7^; Federico Longhini, MD^7^; Angela Quirino, PhD^1^; Giovanni Matera, MD^1^.

^1^Clinical Microbiology Unit, Department of Health Sciences, “Magna Graecia” University, 88100 Catanzaro, Italy; ^2^Microbiology and Virology Unit, "R. Dulbecco" University Hospital, 88100 Catanzaro, Italy; ^3^Clinical Pathology Unit, Department of Health Sciences, University "Magna Græcia" of Catanzaro, 88100 Catanzaro, Italy; ^4^Clinical Microbiology Unit, “Renato Dulbecco” University Hospital, 88100 Catanzaro, Italy; ^5^Microbiology Unit, "Annunziata" Hub Hospital, Azienda Ospedaliera di Cosenza, Cosenza, Italy; ^6^Department of Chemical Sciences, University of Naples Federico II, Naples, Italy; ^7^Anesthesia and Intensive Care, Department of Medical and Surgical Sciences, “Magna Graecia” University, 88100, Catanzaro, Italy.

**Corresponding Author:**

Prof. Federico Longhini

Full Professor in Anaesthesia and Intensive Care

Department of Medical and Surgical Sciences, “Magna Graecia” University

Viale Europa, 88100 Catanzaro, Italy

Tel: +393475395967

**EXTENDED STATISTICAL ANALYSIS PLAN**

Data distribution was assessed using the Shapiro-Wilk test. As biomarker values showed a non-normal distribution with marked right skewness, logarithmic transformation was attempted but did not achieve normality; accordingly, subsequent analyses were conducted on the original scale using non-parametric methods (1-3). Continuous variables are presented as median [interquartile range], and categorical variables as counts (percentages). Given the non-parametric distribution of biomarker values and the repeated-measures design, temporal changes across T0, T1, and T2 were analysed using the Friedman test (4). When the overall test was significant, Dunn’s post hoc multiple-comparison procedure with Bonferroni correction was applied to compare T1 vs T0, T2 vs T0, and T2 vs T1 (5-6). These analyses were performed in the overall cohort and separately according to outcome status (survivors and non-survivors).

Comparisons between survivors and non-survivors at each time point were performed using two-sided Mann-Whitney U tests, and the reported p values were unadjusted. Calprotectin was considered the principal biomarker of interest according to the primary study objective, whereas analyses of the remaining routine biomarkers, cytokines, and growth factors were considered exploratory. To address the potential inflation of type I error arising from multiple testing, the Benjamini-Hochberg procedure was applied to control the false-discovery rate across the 63 secondary biomarker-by-time comparisons, excluding the three comparisons involving calprotectin (7).

For each biomarker, longitudinal changes across the three time points were analysed using a mixed-effects model for repeated measures, with group (survivors vs non-survivors) included as a between-subjects factor and time (T0, T1, T2) as a within-subjects factor. The group × time interaction formally tested whether the longitudinal biomarker trajectory differed between the two outcome groups (8). To account for multiplicity across the 22 trajectory comparisons, the Benjamini-Hochberg procedure was applied, and both unadjusted p values and FDR-adjusted q values were reported.

Effect sizes for statistically significant pairwise comparisons were expressed as median difference (95% confidence interval) (9).

To explore the relationships between baseline calprotectin and complementary clinical and biological measures, two-sided Spearman rank-correlation analyses were performed between calprotectin at T0 and SOFA-2, WBC count, neutrophil count, lymphocyte count, NLR, platelet count, creatinine, bilirubin, CRP and PCT, IL-1α, IL-1β, IL-2, IL-4, IL-6, IL-8, IL-10, IFN-γ, MCP-1 and TNF-α, VEGF, and EGF measured at the same time point. To limit multiplicity, the Benjamini-Hochberg procedure was applied across the 22 correlation tests. Correlations were considered exploratory and were not used for data-driven predictor selection or model specification.

Receiver Operating Characteristic (ROC) curve analysis was performed for SOFA-2 and calprotectin and, on an exploratory basis, for secondary biomarkers showing nominal unadjusted differences between survivors and non-survivors at the corresponding time point. Discriminative performance was quantified by the area under the ROC curve (AUC), with 95% confidence intervals estimated using the DeLong method (10). Exact binomial confidence intervals were calculated for sensitivity, specificity, and predictive values (11). The optimal cut-off value was identified using the Youden index (12). Sensitivity, specificity, positive (PPV) and negative (NPV) predictive value, positive (LR+) and negative (LR-) likelihood ratio were calculated using the observed 28-day mortality prevalence and are therefore cohort-specific (13-15). The AUC of SOFA-2 was compared with that of baseline calprotectin using the DeLong method for correlated ROC curves (10).

Given the limited number of deaths, multivariable analyses were performed using Firth’s bias-reduced penalized logistic regression (16). To limit model overfitting, separate clinically selected parsimonious models were fitted for each baseline biomarker. Each model included age, SOFA-2 score, and one biomarker measured at T0: calprotectin, NLR, CRP, or PCT. Age was expressed per 10-year increase, SOFA-2 per one-point increase, and biomarkers were log2-transformed; therefore, biomarker odds ratios (OR) represent the change in the odds of 28-day mortality associated with a doubling of the biomarker value. No data-driven variable-selection procedure was used.

Model discrimination was quantified using the AUC with 95% confidence intervals (95% CI). Internal validation was performed using 500 stratified bootstrap resamples to estimate optimism-corrected AUCs (17). The independent contribution of each biomarker was evaluated using penalized likelihood-ratio tests. Differences between the apparent AUCs of nested models were evaluated using the DeLong method and were considered exploratory (10).

An additional exploratory multimarker model included age, SOFA-2, calprotectin, NLR, and CRP. Because of the limited number of outcome events relative to the number of predictors, this model was used only to explore whether the combination of biomarkers provided additional discrimination and was not intended as a definitive prediction model.

Given the limited number of outcome events and the large number of measured biomarkers, no data-driven search for optimal biomarker combinations or exhaustive biomarker-correlation network analysis was performed. The analyses instead focused on parsimonious, clinically interpretable models selected according to the study objectives.

All statistical tests were two-sided, with statistical significance set at p<0.05.

**EXTENDED RESULTS**

***Study population***

All 82 included patients had complete measurements at T0, T1, and T2 for calprotectin, routine biomarkers, cytokines, and growth factors. All analyses therefore included 82 patients, comprising 64 survivors and 18 non-survivors, and no statistical imputation was performed.

Age, sex, department of admission, and distribution of the infecting Gram-negative species did not differ significantly between survivors and non-survivors. Non-survivors presented with greater clinical severity, as indicated by a higher SOFA-2 score (median 8 [IQR, 6-11] vs 4 [2-8]; p<0.001) and more frequent use of non-invasive or invasive mechanical ventilation. Complete baseline characteristics and microbiological findings are reported in Table 1 in the main manuscript.

***Longitudinal changes in the overall cohort***

Calprotectin concentrations changed significantly over time (Friedman p<0.001), decreasing from 21.69 [12.30-30.32] ng/mL at T0 to 18.15 [12.15-26.40] ng/mL at T1 and 15.26 [8.16-22.89] ng/mL at T2. Concentrations at T2 were lower than at T0 (p<0.001; median difference, -5.01 ng/mL; 95% CI, -9.76 to -1.89) and T1 (p=0.010; median difference, -1.88 ng/mL; 95% CI, -3.81 to -0.67), whereas the T0-to-T1 comparison was not significant (p=0.057).

WBC count, neutrophil count, NLR, CRP, and PCT also changed significantly over time (all Friedman p<0.001), with lower values at T2 than at both T0 and T1. Lymphocyte count increased between T1 and T2 (p=0.037), whereas platelet count and creatinine were lower at T2 than at T0 (p=0.024 and p=0.030, respectively). Bilirubin did not change significantly over time (p=0.081).

Among cytokines, significant overall temporal changes were observed for IL-1β (p=0.024), IL-2 (p=0.030), IL-4 (p<0.001), IL-6 (p<0.001), IL-10 (p<0.001), IFN-γ (p=0.040), MCP-1 (p<0.001), and TNF-α (p<0.001). IL-1β, IL-2, and IFN-γ were lower at T2 than at T0. IL-4, IL-10, MCP-1, and TNF-α decreased mainly between T0 and T1 and remained lower at T2. IL-6 declined between T0 and T1, T0 and T2, and T1 and T2. IL-1α and IL-8 did not show significant temporal changes. Neither EGF nor VEGF changed significantly in the overall cohort. Complete medians, interquartile ranges, pairwise comparisons, and effect sizes are reported in Table E2.

***Longitudinal changes according to outcome***

Among survivors, calprotectin concentrations decreased from 18.18 [11.27-28.96] ng/mL at T0 to 17.31 [11.49-25.24] ng/mL at T1 and 13.07 [7.80-21.95] ng/mL at T2 (p<0.001). Concentrations at T2 were lower than at T0 (p<0.001) and T1 (p=0.040). Among non-survivors, calprotectin decreased from 30.16 [22.89-39.58] ng/mL at T0 to 23.56 [17.44-31.89] ng/mL at T1 and 22.79 [11.36-28.32] ng/mL at T2 (p=0.001), with a significant reduction between T0 and T2 (p<0.001).

Among survivors, WBC count, neutrophil count, NLR, CRP, and PCT were lower at T2 than at both T0 and T1. Creatinine was also lower at T2 than at T0 and T1, whereas bilirubin showed a smaller reduction between T0 and T2. Platelet and lymphocyte counts did not change significantly. Among non-survivors, WBC, neutrophil and lymphocyte counts, creatinine, bilirubin, and CRP did not change significantly. NLR was lower at T2 than at T1 (p=0.037), platelet count was lower at T2 than at T0 (p=0.014), and PCT was lower at T2 than at T0 (p=0.011).

Among survivors, IL-1β, IL-2, IL-4, IL-6, IL-10, IFN-γ, MCP-1, and TNF-α showed significant temporal reductions. Among non-survivors, overall temporal changes were observed for IL-6, IL-10, and TNF-α. Corrected pairwise comparisons showed lower IL-6 at T2 than at T0 (p=0.037) and lower IL-10 at both T1 (p=0.005) and T2 (p=0.014) than at T0; no individual TNF-α pairwise comparison remained significant after correction. IL-1α, IL-1β, IL-2, IL-4, IL-8, IFN-γ, and MCP-1 did not change significantly among non-survivors.

EGF did not change significantly among survivors but decreased among non-survivors (Friedman p=0.030), with lower concentrations at T2 than at T0 (p=0.037). VEGF did not change significantly in either outcome group. Complete outcome-stratified results are reported in Table E3.

***Time point-specific comparisons between survivors and non-survivors***

Calprotectin was higher in non-survivors than in survivors at all three time points. At T0, median concentrations were 30.16 versus 18.18 ng/mL (p=0.003; median difference, 11.02 ng/mL; 95% CI, 4.30-17.02). Corresponding values were 23.56 versus 17.31 ng/mL at T1 (p=0.037; median difference, 6.25 ng/mL; 95% CI, 0.43-12.40) and 22.79 versus 13.07 ng/mL at T2 (p=0.035; median difference, 6.04 ng/mL; 95% CI, 0.54-11.92).

Among secondary routine biomarkers, non-survivors had nominally higher neutrophil counts at T1 (p=0.034) and T2 (p=0.045), higher NLR at T1 (p=0.047) and T2 (p=0.002), lower lymphocyte counts at T2 (p=0.007), and higher CRP at T2 (p=0.013). The corresponding median differences at T2 were 3.4 × 10³/µL for neutrophils, 8.4 for NLR, -0.3 × 10³/µL for lymphocytes, and 48.0 mg/L for CRP. WBC count, platelet count, creatinine, bilirubin, and PCT did not differ significantly between outcome groups at any time point.

No cytokine differed between outcome groups at T0 or T1. At T2, IL-4 and IL-6 were nominally higher in non-survivors (both p=0.011), with median differences of 1.03 pg/mL and 61 pg/mL, respectively. Differences in MCP-1 (p=0.055), TNF-α (p=0.064), and IL-10 (p=0.076) did not reach nominal statistical significance. Neither EGF nor VEGF differed between outcome groups at any time point.

None of the 63 secondary biomarker-by-time comparisons remained statistically significant after Benjamini-Hochberg false-discovery-rate correction. The lowest adjusted q values were 0.114 for NLR at T2 and 0.167 for lymphocyte count, CRP, IL-4, and IL-6 at T2. Accordingly, all time point-specific findings involving routine biomarkers, cytokines, and growth factors should be considered exploratory. Complete unadjusted p values, adjusted q values, and effect sizes are reported in Table E4.

***Comparison of longitudinal trajectories***

The longitudinal calprotectin trajectory did not differ between survivors and non-survivors (p=0.408), indicating that the decline observed during the first week was not statistically different between outcome groups.

Nominal differences in longitudinal trajectories were identified for WBC count (p=0.040), neutrophil count (p=0.032), NLR (p=0.012), platelet count (p=0.002), bilirubin (p=0.031), and CRP (p=0.039). After Benjamini-Hochberg correction across the 22 trajectory tests, only the platelet trajectory remained statistically different between survivors and non-survivors (q=0.044). The adjusted q values were 0.132 for NLR and 0.147 for WBC count, neutrophil count, bilirubin, and CRP.

No significant trajectory differences were observed for PCT, creatinine, lymphocyte count, any measured cytokine, EGF, or VEGF. Complete model statistics and FDR-adjusted q values are reported in Table E5.

***Exploratory ROC analyses***

SOFA-2 showed strong discrimination for 28-day mortality, with an AUC of 0.897 (95% CI, 0.828-0.965; p<0.001). A cohort-specific threshold of SOFA-2 ≥5 provided 100.0% sensitivity, 60.9% specificity, 41.9% positive predictive value, and 100.0% negative predictive value. SOFA-2 discriminated mortality significantly better than baseline calprotectin (difference in AUC, 0.168; p=0.027).

Calprotectin showed moderate discrimination at T0, with an AUC of 0.728 (95% CI, 0.598-0.858; p<0.001). The threshold >20.54 ng/mL provided 88.9% sensitivity, 57.8% specificity, 37.2% positive predictive value, and 94.9% negative predictive value. At T1, the AUC was 0.662 (95% CI, 0.515-0.810; p=0.031), and at T2 it was 0.664 (95% CI, 0.513-0.815; p=0.033). The corresponding thresholds were >17.92 ng/mL at T1 and >22.60 ng/mL at T2.

Among secondary biomarkers selected on the basis of nominal unadjusted between-group differences, the highest exploratory AUC was observed for NLR at T2 (AUC, 0.742; 95% CI, 0.604-0.880). The AUCs were 0.655 for NLR at T1, 0.665 and 0.656 for neutrophil count at T1 and T2, respectively, 0.708 for lymphocyte count at T2, and 0.692 for CRP at T2. At T2, both IL-4 and IL-6 had an AUC of 0.697. Complete thresholds, sensitivities, specificities, likelihood ratios, and predictive values are reported in Table E6. Because none of the corresponding secondary between-group comparisons remained significant after FDR correction, these ROC findings should be regarded as descriptive and hypothesis-generating.

***Relationships between baseline calprotectin and other measures***

Baseline calprotectin showed FDR-significant positive correlations with neutrophil count (ρ=0.439, p<0.001, q<0.001), WBC count (ρ=0.424, p<0.001, q<0.001), NLR (ρ=0.355, p=0.001, q=0.008), VEGF (ρ=0.294, p=0.007, q=0.040), and CRP (ρ=0.281, p=0.011, q=0.046).

The weaker correlation with SOFA-2 was nominally significant before correction (ρ=0.224, p=0.043) but not after FDR adjustment (q=0.156). No FDR-significant correlations were observed with PCT, lymphocyte or platelet counts, creatinine, bilirubin, IL-1α, IL-1β, IL-2, IL-4, IL-6, IL-8, IL-10, IFN-γ, MCP-1, TNF-α, or EGF. Complete coefficients and adjusted q values are reported in Table E7. These exploratory correlations were not used for predictor selection or model specification.

***Multivariable prognostic models***

The clinical model including age and SOFA-2 had an apparent AUC of 0.899 (95% CI, 0.821-0.978) and an optimism-corrected AUC of 0.893. Both age (OR, 1.81 per 10-year increase; 95% CI, 1.11-3.46; p=0.015) and SOFA-2 (OR, 1.98 per one-point increase; 95% CI, 1.49-2.90; p<0.001) were independently associated with mortality.

After adjustment for age and SOFA-2, baseline calprotectin remained associated with 28-day mortality (OR, 2.07 per doubling; 95% CI, 1.14-4.20; p=0.018). Adding calprotectin increased the apparent AUC to 0.922 (95% CI, 0.854-0.990), with an optimism-corrected AUC of 0.907. However, the improvement over the age-plus-SOFA-2 model was modest and not statistically significant (ΔAUC, 0.023; p=0.117).

Baseline NLR (OR, 1.05 per doubling; 95% CI, 0.66-1.61; p=0.838), CRP (OR, 1.08; 95% CI, 0.57-2.34; p=0.823), and PCT (OR, 0.91; 95% CI, 0.72-1.14; p=0.411) were not independently associated with mortality after adjustment for age and SOFA-2. Their addition did not materially improve discrimination: the apparent AUCs were 0.897 for NLR, 0.904 for CRP, and 0.901 for PCT, with optimism-corrected AUCs of 0.886, 0.889, and 0.886, respectively.

Penalized likelihood-ratio tests for the contribution of each biomarker beyond age and SOFA-2 yielded χ²=5.62 (p=0.018) for calprotectin, χ²=0.04 (p=0.838) for NLR, χ²=0.05 (p=0.823) for CRP, and χ²=0.67 (p=0.411) for PCT.

The exploratory multimarker model including age, SOFA-2, calprotectin, NLR, and CRP had an apparent AUC of 0.920 and an optimism-corrected AUC of 0.898. It therefore provided no evident improvement over the simpler model including age, SOFA-2, and calprotectin. Because only 18 deaths occurred, all multivariable performance estimates and cohort-derived thresholds require external validation. Complete model results are reported in Table 2 in the main manuscript.

**Table E1. Reportable ranges, intra-assay and inter-assay coefficients of variation for the Cytokines Array I High Sensitivity kit**

| **Analyte** | **Range** | **Intra-assay CV (%)** | | | **Inter-assay CV (%)** | | |
| --- | --- | --- | --- | --- | --- | --- | --- |
|  |  | **Level 1** | **Level 2** | **Level 3** | **Level 1** | **Level 2** | **Level 3** |
| IL-1α | 0-225 pg/mL | 10.9 | 9.7 | 11.4 | 15.5 | 8.9 | 13.4 |
| IL-1β | 0-112.5 pg/mL | 9.3 | 8.7 | 7.4 | 9.9 | 11.8 | 8.7 |
| IL-2 | 0-1200 pg/mL | 7.8 | 5.8 | 6.9 | 7.8 | 6.5 | 8.2 |
| IL-4 | 0-450 pg/mL | 9.6 | 9.5 | 8.4 | 11.6 | 8.6 | 8.8 |
| IL-6 | 0-400 pg/mL | 11.9 | 9.8 | 7.8 | 8.4 | 7.4 | 8.4 |
| IL-8 | 0-1450 pg/mL | 9.4 | 9.4 | 7.0 | 11.1 | 9.9 | 9.2 |
| IL-10 | 0-450 pg/mL | 6.8 | 5.6 | 6.1 | 6.7 | 6.5 | 7.5 |
| IFN-γ | 0-600 pg/mL | 10.1 | 7.4 | 7.7 | 11.4 | 6.4 | 7.6 |
| MCP-1 | 0-500 pg/mL | 12.2 | 8.9 | 5.8 | 12.8 | 10.4 | 7.2 |
| TNF-α | 0-600 pg/mL | 7.1 | 7.2 | 12.7 | 8.6 | 6.7 | 7.0 |
| EGF | 0-450 pg/mL | 9.2 | 9.5 | 9.6 | 11.7 | 8.5 | 9.6 |
| VEGF | 0-1000 pg/mL | 10.4 | 10.8 | 7.3 | 12.0 | 7.2 | 10.7 |

Values are reported as coefficient of variation (CV, %) at three assay levels. Precision was evaluated at three assay control levels, reported by the manufacturer as Level 1, Level 2 and Level 3, corresponding to low, intermediate and high concentration ranges, respectively. For each analyte, intra-assay CVs describe variability within the same analytical run, whereas inter-assay CVs describe variability between independent analytical runs.

IL, interleukin; IFN-γ, Interferon gamma; MCP-1, Monocyte Chemoattractant Protein-1; TNF-α, Tumor Necrosis Factor alpha; EGF, Epidermal Growth Factor; VEGF, Vascular Endothelial Growth Factor.

**Table E2. Longitudinal profiles of biomarkers, cytokines and growth factors in the entire population**

|  | | **T0**  **(n=82)** | | | **T1**  **(n=82)** | **T2**  **(n=82)** | **Friedman test** | **Multiple comparisons** | **Effect size**  **Median difference (95%CI)** |  |
| --- | --- | --- | --- | --- | --- | --- | --- | --- | --- | --- |
| *Biomarkers* | | | | | | | | | | |
| *Calprotectin (ng/mL)* | | | 21.69 [12.30; 30.32] | 18.15 [12.15; 26.40] | | 15.26 [8.16; 22.89] | *<0.001* | *a: 0.057*  ***b: <0.001***  ***c: 0.010*** | *b: -5.01 (-9.76 to -1.89)*  *c: -1.88 (-3.81 to -0.67)* |  |
| *WBC (10^3^/μL)* | | | 11.9 [8.5; 17.5] | 11.3 [7.7; 15.7] | | 10.0 [6.8; 13.8] | *<0.001* | *a: 0.515*  ***b: <0.001***  ***c: 0.012*** | *b: -3.1 (-4.4 to -1.8)*  *c: -1.8 (-2.5 to -0.6)* |  |
| *Neutrophils (10^3^/μL)* | | | 10.2 [6.6; 15.8] | 9.2 [6.2; 13.7] | | 8.1 [4.9; 11.8] | *<0.001* | *a: >0.999*  ***b: <0.001***  ***c: 0.003*** | *b: -2.7 (-3.9 to -1.7)*  *c: -1.8 (-2.5 to -0.5)* |  |
| *Lymphocytes (10^3^/μL)* | | | 0.75 [0.39; 1.26] | 0.71 [0.43; 1.07] | | 0.90 [0.54; 1.26] | *0.039* | *a: >0.999*  *b: 0.328*  ***c: 0.037*** | *c: 0.09 (0.01 to 0.24)* |  |
| *NLR* | | | 13.0 [7.1; 30.2] | 13.3 [6.8; 23.6] | | 8.5 [4.5; 16.1] | *<0.001* | *a: 0.446*  ***b: <0.001***  ***c: <0.001*** | *b: -5.3 (-9.5 to -1.6)*  *c: -3.8 (-4.9 to -1.3)* |  |
| *Platelets (10^3^/μL)* | | | 205 [130; 272] | 180 [117; 274] | | 173 [107; 291] | *0.028* | *a: 0.355*  ***b: 0.024***  *c: 0.823* | *b: -18 (-43 to -1)* |  |
| *Creatinine (mg/dL)* | | | 1.20 [0.70; 2.50] | 1.10 [0.60; 1.90] | | 1.10 [0.70; 1.93] | *0.014* | *a: >0.999*  ***b: 0.030***  *c: 0.078* | *b: -0.10 (-0.20 to 0.00)* |  |
| *Bilirubin (mg/dL)* | | | 0.71 [0.40; 1.27] | 0.61 [0.41; 1.01] | | 0.60 [0.31; 0.85] | *0.081* | *n.a.* |  |  |
| *CRP (mg/L)* | | | 154 [100; 196] | 127 [84; 172] | | 82 [26; 127] | *<0.001* | *a: 0.593*  ***b: <0.001***  ***c: <0.001*** | *b: -61 (-97 to -37)*  *c: -39 (-57 to -20)* |  |
| *PCT (ng/mL)* | | | 6.7 [1.1; 35.8] | 4.3 [1.0; 17.0] | | 1.0 [0.3; 3.9] | *<0.001* | *a: 0.280*  ***b: <0.001***  ***c: <0.001*** | *b: -3.0 (-9.0 to -0.9)*  *c: -2.8 (-4.1 to -1.8)* |  |
| *Cytokines* | | | | | | | | | | |
| *IL-1α (pg/mL)* | *0.53 [0.37; 0.81]* | | | 0.51 [0.37; 0.81] | | 0.47 [0.32; 0.65] | *0.280* | *n.a.* |  |  |
| *IL-1β (pg/mL)* | *3.87 [2.25; 7.70]* | | | 2.71 [2.08; 4.74] | | 2.56 [1.62; 4.58] | *0.024* | *a: 1.000*  ***b: 0.023***  *c: 0.212* | *b: -0.53 (-0.95 to -0.12)* |  |
| *IL-2 (pg/mL)* | *1.17 [0.85; 2.39]* | | | 1.16 [0.70; 1.85] | | 1.02 [0.75; 1.46] | *0.030* | *a: 0.230*  ***b: 0.028***  *c: 1.000* | *b: -0.18 (-0.49 to 0.00)* |  |
| *IL-4 (pg/mL)* | *3.38 [2.00; 5.81]* | | | 1.73 [1.18; 3.07] | | 1.94 [1.25; 2.85] | *<0.001* | ***a: <0.001***  ***b: <0.001***  *c: 0.714* | *a: -1.11 (-1.97 to -0.60)*  *b: -1.06 (-1.69 to -0.71)* |  |
| *IL-6 (pg/mL)* | *264 [105; 732]* | | | 82 [28; 140] | | 42 [19; 125] | *<0.001* | ***a: <0.001***  ***b: <0.001***  ***c: 0.018*** | *a: -120 (-216 to -41)*  *b: -127 (-283 to -86)*  *c: -6 (-18 to -3)* |  |
| *IL-8 (pg/mL)* | *818 [192; 818]* | | | 434 [137; 818] | | 328 [153; 818] | *0.122* | *n.a.* |  |  |
| *IL-10 (pg/mL)* | *14.69 [2.76; 164.12]* | | | 3.42 [1.26; 8.94] | | 2.94 [1.26; 7.75] | *<0.001* | ***a: <0.001***  ***b: <0.001***  *c: 0.668* | *a: -7.86 (-15.12 to -2.41)*  *b: -6.16 (-17.60 to -2.22)* |  |
| *IFN-γ (pg/mL)* | *1.64 [0.54; 4.99]* | | | 1.01 [0.47; 2.79] | | 0.68 [0.40; 1.84] | *0.040* | *a: 0.210*  ***b: 0.044***  *c: 1.000* | *b: -0.21 (-0.64 to 0.00)* |  |
| *MCP-1 (pg/mL)* | *723 [421; 843]* | | | 475 [343; 781] | | 402 [267; 816] | *<0.001* | ***a: 0.007***  ***b: 0.002***  *c: 1.000* | *a: -88 (-218 to 0)*  *b: -107 (-219 to -21)* |  |
| *TNF-α (pg/mL)* | *14.06 [6.08; 32.17]* | | | 7.99 [4.47; 15.59] | | 6.25 [4.36; 13.11] | *<0.001* | ***a: <0.001***  ***b: <0.001***  *c: 1.000* | *a: -3.47 (-7.54 to -1.42)*  *b: -3.97 (-7.61 to -1.14)* |  |
| *Growth Factors* | | | | | | | | | | |
| *EGF (pg/mL)* | *109 [56; 185]* | | | 114 [70; 156] | | 108 [71; 197] | *0.236* | *n.a.* |  |  |
| *VEGF (pg/mL)* | *254 [106; 450]* | | | 239 [107; 539] | | 241 [138; 580] | *0.618* | *n.a.* |  |  |

WBC, White Blood Cell count; NLR, Neutrophils to Lymphocytes Ratio; CRP, C-reactive protein; PCT, Procalcitonin; IL, interleukin; IFN-γ, Interferon gamma; MCP-1, Monocyte Chemoattractant Protein-1; TNF-α, Tumor Necrosis Factor alpha; EGF, Epidermal Growth Factor; VEGF, Vascular Endothelial Growth Factor. a, T1 vs. T0; b, T2 vs. T0; c, T2 vs. T1; n.a., not applicable.

**Table E3. Longitudinal profiles of biomarkers, cytokines and growth factors according to outcome**

|  | | **T0** | **T1** | **T2** | **Friedman test** | **Multiple comparisons** | **Effect size**  **Median difference (95%CI)** |  |
| --- | --- | --- | --- | --- | --- | --- | --- | --- |
| ***Biomarkers*** | | | | | | | | |
| ***Calprotectin (ng/mL)*** |  | |  |  |  |  |  |  |
| *Survivors (n=64)* | 18.18 [11.27; 28.96] | | 17.31 [11.49; 25.24] | 13.07 [7.80; 21.95] | *<0.001* | *a: 0.472*  ***b: <0.001***  ***c: 0.040*** | *b: -4.10 (-9.29 to -0.68)*  *c: -1.88 (-4.57 to -0.67)* |  |
| *Non-survivors (n=18)* | 30.16 [22.89; 39.58] | | 23.56 [17.44; 31.89] | 22.79 [11.36; 28.32] | *0.001* | *a: 0.059*  ***b: <0.001***  *c: 0.547* | *b: -10.43 (-16.44 to -0.99)* |  |
| ***WBC (10^3^/μL)*** |  | |  |  |  |  |  |  |
| *Survivors (n=64)* | 11.6 [8.5; 16.7] | | 11.0 [7.5; 15.2] | 9.4 [5.8; 13.5] | *<0.001* | *a: 0.434*  ***b: <0.001***  ***c: 0.024*** | *b: -3.5 (-5.0 to -1.8)*  *c: -2.0 (-2.8 to -1.0)* |  |
| *Non-survivors (n=18)* | 13.9 [7.8; 22.7] | | 13.9 [8.8; 28.5] | 11.3 [8.3; 23.4] | *0.348* |  |  |  |
| ***Neutrophils (10^3^/μL)*** |  | |  |  |  |  |  |  |
| *Survivors (n=64)* | 9.9 [6.6; 13.8] | | 8.8 [6.1; 12.1] | 7.7 [4.4; 11.7] | *<0.001* | *a: 0.928*  ***b: <0.001***  ***c: 0.008*** | *b: -2.8 (-4.0 to -1.7)*  *c: -1.8 (-2.9 to -0.5)* |  |
| *Non-survivors (n=18)* | 12.6 [6.6; 20.0] | | 12.3 [7.1; 26.6] | 9.7 [7.6; 22.2] | *0.223* |  |  |  |
| ***Lymphocytes (10^3^/μL)*** |  | |  |  |  |  |  |  |
| *Survivors (n=64)* | 0.8 [0.4; 1.4] | | 0.8 [0.5; 1.1] | 1.0 [0.6; 1.4] | *0.106* |  |  |  |
| *Non-survivors (n=18)* | 0.7 [0.3; 0.9] | | 0.6 [0.3; 1.0] | 0.6 [0.5; 0.9] | *0.358* |  |  |  |
| ***NLR*** |  | |  |  |  |  |  |  |
| *Survivors (n=64)* | 12.0 [6.5; 30.9] | | 11.4 [6.5; 20.0] | 7.3 [3.7; 13.1] | *<0.001* | *a: 0.091*  ***b: <0.001***  ***c: 0.006*** | *b: -5.0 (-14.0 to -1.3)*  *c: -4.0 (-6.5 to -1.2)* |  |
| *Non-survivors (n=18)* | 19.4 [10.9; 31.6] | | 20.0 [7.2; 41.0] | 16.7 [8.8; 30.4] | *0.042* | *a: 0.952*  *b: 0.401*  ***c: 0.037*** | *c: -1.6 (-12.6 to -0.2)* |  |
| ***Platelets (10^3^/μL)*** |  | |  |  |  |  |  |  |
| *Survivors (n=64)* | 192 [130; 271] | | 181 [117; 275] | 178 [111; 302] | *0.288* |  |  |  |
| *Non-survivors (n=18)* | 250 [174; 309] | | 180 [117; 259] | 137 [85; 242] | *0.017* | *a: 0.730*  ***b: 0.014***  *c: 0.287* | *b: -83 (-127 to -6)* |  |
| ***Creatinine (mg/dL)*** |  | |  |  |  |  |  |  |
| *Survivors (n=64)* | 1.50 [0.70; 2.57] | | 1.25 [0.70; 2.13] | 1.10 [0.70; 1.98] | *0.003* | *a: >0.999*  ***b: 0.007***  ***c: 0.035*** | *b: -0.1 (-0.2 to -0.1)*  *c: -0.1 (-0.2 to 0.0)* |  |
| *Non-survivors (n=18)* | 0.95 [0.70; 1.68] | | 0.90 [0.40; 1.33] | 1.15 [0.58; 1.95] | *0.956* |  |  |  |
| ***Bilirubin (mg/dL)*** |  | |  |  |  |  |  |  |
| *Survivors (n=64)* | 0.70 [0.39; 1.25] | | 0.61 [0.40; 0.90] | 0.59 [0.31; 0.80] | *0.034* | *a: 0.928*  ***b: 0.031***  *c: 0.366* |  |  |
| *Non-survivors (n=18)* | 0.78 [0.47; 1.44] | | 0.66 [0.47; 1.47] | 0.66 [0.30; 1.50] | *0.945* |  |  |  |
| ***CRP (mg/L)*** |  | |  |  |  |  |  |  |
| *Survivors (n=64)* | 149 [104; 198] | | 127 [83; 169] | 64 [25; 115] | *<0.001* | *a: 0.600*  ***b: <0.001***  ***c: <0.001*** | *b: -66 (-130 to -40)*  *c: -40 (-61 to -20)* |  |
| *Non-survivors (n=18)* | 171 [85; 191] | | 137 [83; 197] | 106 [73; 183] | *0.211* | *n.a.* |  |  |
| ***PCT (ng/mL)*** |  | |  |  |  |  |  |  |
| *Survivors (n=64)* | 7.6 [1.8; 36.3] | | 4.5 [1.0; 16.5] | 0.9 [0.3; 3.7] | *<0.001* | *a: >0.999*  ***b: <0.001***  ***c: <0.001*** | *b: -3.0 (-11.0 to -0.9)*  *c: -2.9 (-4.8 to -1.9)* |  |
| *Non-survivors (n=18)* | 4.4 [0.4; 30.0] | | 3.1 [0.3; 18.2] | 1.4 [0.6; 8.2] | *0.009* | *a: 0.117*  ***b: 0.011***  *c: >0.999* | *b: -3.6 (-18.4 to -0.1)* |  |
|  |  | |  |  |  |  |  |  |
| ***Cytokines*** | | | | | | | |  |
| ***IL-1α (pg/mL)*** |  | |  |  |  |  |  |  |
| *Survivors (n=64)* | 0.54 [0.41; 0.84] | | 0.50 [0.37; 0.83] | 0.49 [0.32; 0.66] | *0.246* | *n.a.* |  |  |
| *Non-survivors (n=18)* | 0.44 [0.32; 0.67] | | 0.53 [0.38; 0.71] | 0.46 [0.33; 0.58] | *0.389* | *n.a.* |  |  |
| ***IL-1β (pg/mL)*** |  | |  |  |  |  |  |  |
| *Survivors (n=64)* | 3.78 [2.18; 5.44] | | 2.61 [2.07; 4.90] | 2.32 [1.54; 3.96] | *0.021* | *a: 1.000*  ***b: 0.038***  *c: 0.062* | *b: -0.46 (-0.92 to -0.08)* |  |
| *Non-survivors (n=18)* | 5.77 [2.60; 11.95] | | 3.38 [2.31; 4.38] | 3.01 [2.62; 5.11] | *0.311* | *n.a.* |  |  |
| ***IL-2 (pg/mL)*** |  | |  |  |  |  |  |  |
| *Survivors (n=64)* | 1.21 [0.86; 2.95] | | 1.16 [0.74; 1.84] | 1.02 [0.75; 1.32] | *0.010* | *a: 0.499*  ***b: 0.007***  *c: 0.296* | *b: -0.26 (-0.55 to -0.04)* |  |
| *Non-survivors (n=18)* | 1.05 [0.82; 1.57] | | 1.12 [0.67; 1.79] | 1.19 [0.75; 3.74] | *0.348* | *n.a.* |  |  |
| ***IL-4 (pg/mL)*** |  | |  |  |  |  |  |  |
| *Survivors (n=64)* | 3.48 [2.02; 5.62] | | 1.66 [1.18; 3.13] | 1.66 [1.21; 2.59] | *<0.001* | ***a: 0.001***  ***b: <0.001***  *c: 0.061* | *a: -0.94 (-1.99 to -0.47)*  *b: -1.31 (-1.98 to -0.76)* |  |
| *Non-survivors (n=18)* | 3.21 [2.00; 6.49] | | 2.09 [1.15; 2.96] | 2.81 [1.78; 5.73] | *0.066* | *n.a.* |  |  |
| ***IL-6 (pg/mL)*** |  | |  |  |  |  |  |  |
| *Survivors (n=64)* | 240 [103; 732] | | 78 [27; 124] | 38 [15; 87] | *<0.001* | ***a: <0.001***  ***b: <0.001***  ***c: 0.017*** | *a: -129 (-286 to -45)*  *b: -146 (-391 to -86)*  *c: -10 (-30 to -3)* |  |
| *Non-survivors (n=18)* | 265 [111; 732] | | 98 [46; 235] | 116 [45; 502] | *0.033* | *a: 0.172*  ***b: 0.037***  *c: 1.000* | *b: -101 (-222 to -10)* |  |
| ***IL-8 (pg/mL)*** |  | |  |  |  |  |  |  |
| *Survivors (n=64)* | 764 [210; 818] | | 578 [117; 818] | 310 [115; 818] | *0.073* | *n.a.* |  |  |
| *Non-survivors (n=18)* | 818 [103; 818] | | 300 [158; 730] | 431 [201; 818] | *0.937* |  |  |  |
| ***IL-10 (pg/mL)*** |  | |  |  |  |  |  |  |
| *Survivors (n=64)* | 14.69 [2.46; 161.28] | | 3.42 [1.53; 8.10] | 2.77 [1.20; 5.39] | *<0.001* | ***a: 0.003***  ***b: <0.001***  *c: 0.355* | *a: -7.86 (-15.11 to -1.27)*  *b: -8.39 (-17.97 to -2.11)* |  |
| *Non-survivors (n=18)* | 12.78 [3.29; 194.05] | | 2.91 [1.26; 9.12] | 6.58 [1.91; 31.03] | *0.002* | ***a: 0.005***  ***b: 0.014***  *c: 1.000* | *a: -7.05 (-130.22 to -1.62) \| b: -3.48 (-168.27 to -0.66)* |  |
| ***IFN-γ (pg/mL)*** |  | |  |  |  |  |  |  |
| *Survivors (n=64)* | 1.78 [0.52; 5.14] | | 1.01 [0.54; 2.92] | 0.65 [0.41; 1.81] | *0.017* | *a: 0.457*  ***b: 0.013***  *c: 0.457* | *b: -0.30 (-0.80 to -0.03)* |  |
| *Non-survivors (n=18)* | 1.00 [0.58; 3.90] | | 1.03 [0.45; 1.80] | 1.04 [0.35; 10.90] | *0.348* | *n.a.* |  |  |
| ***MCP-1 (pg/mL)*** |  | |  |  |  |  |  |  |
| *Survivors (n=64)* | 794 [484; 843] | | 475 [354; 738] | 347 [251; 718] | *<0.001* | ***a: 0.009***  ***b: <0.001***  *c: 0.774* | *a: -99 (-245 to 0)*  *b: -138 (-275 to -42)* |  |
| *Non-survivors (n=18)* | 546 [337; 843] | | 477 [309; 824] | 600 [372; 843] | *0.455* | *n.a.* |  |  |
| ***TNF-α (pg/mL)*** |  | |  |  |  |  |  |  |
| *Survivors (n=64)* | 13.07 [6.11; 31.73] | | 7.84 [4.29; 13.52] | 5.94 [4.31; 10.29] | *<0.001* | ***a: 0.004***  ***b: <0.001***  *c: 1.000* | *a: -3.29 (-7.76 to -0.94) \| b: -2.63 (-8.46 to -0.63)* |  |
| *Non-survivors (n=18)* | 15.79 [6.29; 31.98] | | 11.96 [5.78; 19.62] | 11.83 [5.51; 18.64] | *0.034* | *a: 0.091*  *b: 0.059*  *c: 1.000* |  |  |
| ***Growth Factors*** | | | | | | | |  |
| ***EGF (pg/mL)*** |  | |  |  |  |  |  |  |
| *Survivors (n=64)* | 108 [56; 170] | | 124 [72; 156] | 112 [81; 212] | *0.276* | *n.a.* |  |  |
| *Non-survivors (n=18)* | 152 [66; 233] | | 96 [60; 133] | 84 [16; 161] | *0.030* | *a: 0.137*  ***b: 0.037***  *c: 1.000* | *b: -63 (-109 to -23)* |  |
| ***VEGF (pg/mL)*** |  | |  |  |  |  |  |  |
| *Survivors (n=64)* | 237 [91; 442] | | 248 [109; 541] | 244 [147; 580] | *0.095* |  |  |  |
| *Non-survivors (n=18)* | 357 [184; 510] | | 273 [103; 535] | 199 [58; 536] | *0.092* |  |  |  |

WBC, White Blood Cell count; NLR, Neutrophils to Lymphocytes Ratio; CRP, C-reactive protein; PCT, Procalcitonin; IL, interleukin; IFN-γ, Interferon gamma; MCP-1, Monocyte Chemoattractant Protein-1; TNF-α, Tumor Necrosis Factor alpha; EGF, Epidermal Growth Factor; VEGF, Vascular Endothelial Growth Factor. a, T1 vs. T0; b, T2 vs. T0; c, T2 vs. T1; n.a., not applicable.

**Table E4. Time point-specific comparisons between survivors and non-survivors, with false-discovery-rate-adjusted q values**

|  | **Survivors**  **(n=64)** | **Non-survivors**  **(n=18)** | **Mann-Whitney U-test (unadjusted p value)** | **FDR-adjusted q value** | **Effect size Hodges-Lehmann median difference (95% CI)** |
| --- | --- | --- | --- | --- | --- |
| ***Biomarkers*** | | | | | |
| ***Calprotectin (ng/mL)*** | | | | | |
| T0 | 18.18 [11.27; 28.96] | 30.16 [22.89; 39.58] | **0.003** | — | 11.02 [4.30 to 17.02] |
| T1 | 17.31 [11.49; 25.24] | 23.56 [17.44; 31.89] | **0.037** | — | 6.25 [0.43 to 12.40] |
| T2 | 13.07 [7.79; 21.95] | 22.79 [11.36; 28.32] | **0.035** | — | 6.04 [0.54 to 11.92] |
| ***WBC (10^3^/μL)*** | | | | | |
| T0 | 11.6 [8.5; 16.5] | 13.9 [9.3; 21.8] | 0.290 | 0.537 |  |
| T1 | 11.0 [7.5; 15.0] | 13.9 [9.3; 26.7] | 0.056 | 0.327 |  |
| T2 | 9.4 [6.1; 13.3] | 11.3 [8.6; 22.5] | 0.084 | 0.376 |  |
| ***Neutrophils (10^3^/μL)*** | | | | | |
| T0 | 9.9 [6.6; 13.6] | 12.6 [7.8; 19.0] | 0.260 | 0.529 |  |
| T1 | 8.8 [6.1; 12.1] | 12.3 [7.9; 25.3] | **0.034** | 0.327 | 4.4 [0.2 to 9.9] |
| T2 | 7.6 [4.4; 11.6] | 9.7 [7.7; 21.0] | **0.045** | 0.327 | 3.4 [0.1 to 7.3] |
| ***Lymphocytes (10^3^/μL)*** | | | | | |
| T0 | 0.8 [0.4; 1.3] | 0.6 [0.3; 0.9] | 0.226 | 0.513 |  |
| T1 | 0.8 [0.5; 1.1] | 0.6 [0.3; 0.9] | 0.126 | 0.471 |  |
| T2 | 0.9 [0.6; 1.4] | 0.6 [0.5; 0.8] | **0.007** | 0.167 | -0.3 [-0.5 to -0.1] |
| ***NLR*** | | | | | |
| T0 | 12.0 [6.4; 30.2] | 19.4 [11.8; 26.9] | 0.220 | 0.513 |  |
| T1 | 11.4 [6.8; 19.9] | 20.0 [8.8; 31.5] | **0.047** | 0.327 | 7.8 [0.1 to 16.6] |
| T2 | 7.3 [3.9; 13.1] | 16.7 [10.2; 28.7] | **0.002** | 0.114 | 8.4 [3.1 to 13.5] |
| ***Platelets (10^3^/μL)*** | | | | | |
| T0 | 192 [130; 270] | 250 [184; 294] | 0.208 | 0.513 |  |
| T1 | 180 [120; 275] | 180 [119; 254] | 0.969 | 0.984 |  |
| T2 | 178 [113; 300] | 136 [93; 230] | 0.156 | 0.493 |  |
| ***Creatinine (mg/dL)*** | | | | | |
| T0 | 1.5 [0.7; 2.5] | 0.9 [0.7; 1.3] | 0.228 | 0.513 |  |
| T1 | 1.2 [0.7; 2.0] | 0.9 [0.4; 1.3] | 0.057 | 0.327 |  |
| T2 | 1.1 [0.7; 1.9] | 1.1 [0.7; 1.8] | 0.707 | 0.874 |  |
| ***Bilirubin (mg/dL)*** | | | | | |
| T0 | 0.7 [0.4; 1.2] | 0.8 [0.5; 1.4] | 0.436 | 0.677 |  |
| T1 | 0.6 [0.4; 0.9] | 0.7 [0.5; 1.3] | 0.394 | 0.664 |  |
| T2 | 0.6 [0.3; 0.8] | 0.7 [0.3; 1.3] | 0.400 | 0.664 |  |
| ***CRP (mg/L)*** | | | | | |
| T0 | 149 [105; 197] | 171 [91; 189] | 0.831 | 0.952 |  |
| T1 | 126 [84; 168] | 137 [86; 180] | 0.595 | 0.791 |  |
| T2 | 64 [25; 112] | 106 [77; 171] | **0.013** | 0.167 | 48.0 [10.2 to 80.3] |
| ***PCT (ng/mL)*** | | | | | |
| T0 | 7.6 [1.8; 36.3] | 4.4 [0.4; 25.4] | 0.290 | 0.537 |  |
| T1 | 4.5 [1.2; 15.6] | 3.1 [0.5; 17.1] | 0.545 | 0.781 |  |
| T2 | 0.8 [0.3; 3.6] | 1.4 [0.7; 6.5] | 0.246 | 0.529 |  |
| ***Cytokines*** | | | | | |
| ***IL-1α (pg/mL)*** | | | | | |
| T0 | 0.54 [0.41; 0.84] | 0.44 [0.32; 0.67] | 0.279 | 0.537 |  |
| T1 | 0.50 [0.37; 0.83] | 0.53 [0.38; 0.71] | 0.933 | 0.984 |  |
| T2 | 0.49 [0.32; 0.66] | 0.46 [0.33; 0.58] | 0.897 | 0.984 |  |
| ***IL-1β (pg/mL)*** | | | | | |
| T0 | 3.78 [2.18; 5.44] | 5.77 [2.60; 11.95] | 0.165 | 0.494 |  |
| T1 | 2.61 [2.07; 4.90] | 3.38 [2.31; 4.38] | 0.538 | 0.781 |  |
| T2 | 2.32 [1.54; 3.96] | 3.01 [2.62; 5.11] | 0.135 | 0.471 |  |
| ***IL-2 (pg/mL)*** | | | | | |
| T0 | 1.21 [0.86; 2.95] | 1.05 [0.82; 1.57] | 0.349 | 0.611 |  |
| T1 | 1.16 [0.74; 1.84] | 1.12 [0.67; 1.79] | 0.630 | 0.794 |  |
| T2 | 1.02 [0.75; 1.32] | 1.19 [0.75; 3.74] | 0.260 | 0.529 |  |
| ***IL-4 (pg/mL)*** | | | | | |
| T0 | 3.48 [2.02; 5.62] | 3.21 [2.00; 6.49] | 0.595 | 0.791 |  |
| T1 | 1.66 [1.18; 3.13] | 2.09 [1.15; 2.96] | 0.805 | 0.951 |  |
| T2 | 1.66 [1.21; 2.59] | 2.81 [1.78; 5.73] | **0.011** | 0.167 | 1.03 [0.20 to 2.05] |
| ***IL-6 (pg/mL)*** | | | | | |
| T0 | 240 [103; 732] | 265 [111; 732] | 0.968 | 0.984 |  |
| T1 | 78 [27; 124] | 98 [46; 235] | 0.200 | 0.513 |  |
| T2 | 38 [15; 87] | 116 [45; 502] | **0.011** | 0.167 | 61 [8 to 120] |
| ***IL-8 (pg/mL)*** | | | | | |
| T0 | 764 [210; 818] | 818 [103; 818] | 0.815 | 0.951 |  |
| T1 | 578 [117; 818] | 300 [158; 730] | 0.571 | 0.791 |  |
| T2 | 310 [115; 818] | 431 [201; 818] | 0.440 | 0.677 |  |
| ***IL-10 (pg/mL)*** | | | | | |
| T0 | 14.69 [2.46; 161.28] | 12.78 [3.29; 194.05] | 0.906 | 0.984 |  |
| T1 | 3.42 [1.53; 8.10] | 2.91 [1.26; 9.12] | 1.000 | 1.000 |  |
| T2 | 2.77 [1.20; 5.39] | 6.58 [1.91; 31.03] | 0.076 | 0.367 |  |
| ***IFN-γ (pg/mL)*** | | | | | |
| T0 | 1.78 [0.52; 5.14] | 1.00 [0.58; 3.90] | 0.788 | 0.951 |  |
| T1 | 1.01 [0.54; 2.92] | 1.03 [0.45; 1.80] | 0.538 | 0.781 |  |
| T2 | 0.65 [0.41; 1.81] | 1.04 [0.35; 10.90] | 0.622 | 0.794 |  |
| ***MCP-1 (pg/mL)*** | | | | | |
| T0 | 794 [484; 843] | 546 [337; 843] | 0.145 | 0.479 |  |
| T1 | 475 [354; 738] | 477 [309; 824] | 0.946 | 0.984 |  |
| T2 | 347 [251; 718] | 600 [372; 843] | 0.055 | 0.327 |  |
| ***TNF-α (pg/mL)*** | | | | | |
| T0 | 13.07 [6.11; 31.73] | 15.79 [6.29; 31.98] | 0.602 | 0.791 |  |
| T1 | 7.84 [4.29; 13.52] | 11.96 [5.78; 19.62] | 0.196 | 0.513 |  |
| T2 | 5.94 [4.31; 10.29] | 11.83 [5.51; 18.64] | 0.064 | 0.334 |  |
| ***Growth factors*** | | | | | |
| ***EGF (pg/mL)*** | | | | | |
| T0 | 108 [56; 170] | 152 [66; 233] | 0.311 | 0.559 |  |
| T1 | 124 [72; 156] | 96 [60; 133] | 0.206 | 0.513 |  |
| T2 | 112 [81; 212] | 84 [16; 161] | 0.090 | 0.376 |  |
| ***VEGF (pg/mL)*** | | | | | |
| T0 | 237 [91; 442] | 357 [184; 510] | 0.132 | 0.471 |  |
| T1 | 248 [109; 541] | 273 [103; 535] | 0.853 | 0.960 |  |
| T2 | 244 [147; 580] | 199 [58; 536] | 0.423 | 0.677 |  |

WBC, White Blood Cell count; NLR, Neutrophils to Lymphocytes Ratio; CRP, C-reactive protein; PCT, Procalcitonin; IL, interleukin; IFN-γ, Interferon gamma; MCP-1, Monocyte Chemoattractant Protein-1; TNF-α, Tumor Necrosis Factor alpha; EGF, Epidermal Growth Factor; VEGF, Vascular Endothelial Growth Factor.

**Table E5. Comparison of biomarker longitudinal kinetics between survivors and non-survivors**

|  | | | **Degrees of freedom** | | **F value** | **P value** | | **FDR-adjusted**  **q value** | |  |
| --- | --- | --- | --- | --- | --- | --- | --- | --- | --- | --- |
|  | ***Biomarkers*** | | | | | | | | | |
| ***Calprotectin (ng/mL)*** | | | | 2 | 0.901 | 0.408 | | 0.816 | |  |
| ***WBC (10^3^/μL)*** | | | | 2 | 3.289 | 0.040 | | 0.147 | |  |
| ***Neutrophils (10^3^/μL)*** | | | | 2 | 3.528 | 0.032 | | 0.147 | |  |
| ***Lymphocytes (10^3^/μL)*** | | | | 2 | 0.176 | 0.839 | | 0.992 | |  |
| ***NLR*** | | | | 2 | 4.563 | 0.012 | | 0.132 | |  |
| ***Platelets (10^3^/μL)*** | | | | 2 | 6.311 | 0.002 | | 0.044 | |  |
| ***Creatinine (mg/dL)*** | | | | 2 | 2.659 | 0.073 | | 0.229 | |  |
| ***Bilirubin (mg/dL)*** | | | | 2 | 3.541 | 0.031 | | 0.147 | |  |
| ***CRP (mg/L)*** | | | | 2 | 3.304 | 0.039 | | 0.147 | |  |
| ***PCT (ng/mL)*** | | | | 2 | 0.671 | 0.513 | | 0.940 | |  |
| ***Cytokines*** | | | | | | |  | |  |  |
| ***IL-1α (pg/mL)*** | | 2 | | | 0.146 | 0.864 | | 0.992 | |  |
| ***IL-1β (pg/mL)*** | | 2 | | | 0.122 | 0.885 | | 0.992 | |  |
| ***IL-2 (pg/mL)*** | | 2 | | | 0.316 | 0.730 | | 0.992 | |  |
| ***IL-4 (pg/mL)*** | | 2 | | | 0.936 | 0.394 | | 0.816 | |  |
| ***IL-6 (pg/mL)*** | | 2 | | | 0.381 | 0.684 | | 0.992 | |  |
| ***IL-8 (pg/mL)*** | | 2 | | | 0.008 | 0.992 | | 0.992 | |  |
| ***IL-10 (pg/mL)*** | | 2 | | | 0.076 | 0.927 | | 0.992 | |  |
| ***IFN-γ (pg/mL)*** | | 2 | | | 1.128 | 0.326 | | 0.797 | |  |
| ***MCP-1 (pg/mL)*** | | 2 | | | 0.040 | 0.961 | | 0.992 | |  |
| ***TNF-α (pg/mL)*** | | 2 | | | 1.285 | 0.280 | | 0.770 | |  |
|  | ***Growth Factors*** | | | | | | | | | |
| ***EGF (pg/mL)*** | | 2 | | | 0.394 | 0.675 | | 0.992 | |  |
| ***VEGF (pg/mL)*** | | 2 | | | 0.444 | 0.642 | | 0.992 | |  |

All trajectory analyses included the complete cohort of 82 patients (64 survivors and 18 non-survivors). WBC, White Blood Cell count; NLR, Neutrophils to Lymphocytes Ratio; CRP, C-reactive protein; PCT, Procalcitonin; IL, interleukin; IFN-γ, Interferon gamma; MCP-1, Monocyte Chemoattractant Protein-1; TNF-α, Tumor Necrosis Factor alpha; EGF, Epidermal Growth Factor; VEGF, Vascular Endothelial Growth Factor.

**Table E6. Receiver Operating Characteristic (ROC) curves analysis for death prediction**

|  | **AUC** | **P value** | **Criterion** | **Sensitivity** | **Specificity** | **LR+** | **LR-** | **PPV** | **NPV** |
| --- | --- | --- | --- | --- | --- | --- | --- | --- | --- |
| ***Biomarkers*** | | | | | | | | | |
| ***Calprotectin (ng/mL)*** | | | | | | | | | |
| *T0* | 0.728  (0.598 to 0.858) | <0.001 | >20.54 | 88.9  (65.3 to 98.6) | 57.8  (44.8 to 70.1) | 2.11  (1.51 to 2.93) | 0.19  (0.05 to 0.72) | 37.2  (23.0 to 53.3) | 94.9  (82.7 to 99.4) |
| *T1* | 0.662  (0.515 to 0.810) | 0.031 | >17.92 | 77.8  (52.4 to 93.6) | 54.7  (41.7 to 67.2) | 1.72  (1.19 to 2.47) | 0.41  (0.17 to 0.99) | 32.6  (19.1 to 48.5) | 89.7  (75.8 to 97.1) |
| *T2* | 0.664  (0.513 to 0.815) | 0.033 | >22.60 | 55.6  (30.8 to 78.5) | 79.7  (67.8 to 88.7) | 2.74  (1.45 to 5.17) | 0.56  (0.33 to 0.95) | 43.5  (23.2 to 65.5) | 86.4  (75.0 to 94.0) |
|  | | | | | | | | | |
| ***Neutrophils (10^3^/μL)*** | | | | | | | | | |
| *T1* | 0.665  (0.498 to 0.832) | 0.053 | >20.34 | 38.9  (17.3 to 64.3) | 92.2  (82.7 to 97.4) | 4.98  (1.79 to 13.83) | 0.66  (0.46 to 0.96) | 58.3  (27.7 to 84.8) | 84.3  (73.6 to 91.9) |
| *T2* | 0.656  (0.501 to 0.811) | 0.048 | >7.29 | 83.3  (58.6 to 96.4) | 46.9  (34.3 to 59.8) | 1.57  (1.15 to 2.14) | 0.36  (0.12 to 1.03) | 30.6  (18.3 to 45.4) | 90.9  (75.7 to 98.1) |
|  |  |  |  |  |  |  |  |  |  |
| ***Lymphocytes (10^3^/μL)*** | | | | | | | | | |
| *T2* | 0.708  (0.580 to 0.836) | 0.001 | <0.58 | 61.1  (35.7 to 82.7) | 78.1  (66.0 to 87.5) | 2.79  (1.55 to 5.05) | 0.50  (0.27 to 0.90) | 44.0  (24.4 to 65.1) | 87.7  (76.3 to 94.9) |
|  |  |  |  |  |  |  |  |  |  |
| ***NLR*** | | | | | | | | | |
| *T1* | 0.655  (0.496 to 0.813) | 0.056 | >15.25 | 66.7  (41.0 to 86.7) | 64.1  (51.1 to 75.7) | 1.86  (1.17 to 2.95) | 0.52  (0.26 to 1.03) | 34.3  (19.1 to 52.2) | 87.2  (74.3 to 95.2) |
| *T2* | 0.742  (0.604 to 0.880) | <0.001 | >12.80 | 72.2  (46.5 to 90.3) | 73.4  (60.9 to 83.7) | 2.72  (1.65 to 4.47) | 0.38  (0.18 to 0.81) | 43.3  (25.5 to 62.6) | 90.4  (79.0 to 96.8) |
|  |  |  |  |  |  |  |  |  |  |
| ***CRP (mg/L)*** | | | | | | | | | |
| *T2* | 0.692  (0.558 to 0.826) | 0.005 | >66.00 | 83.3  (58.6 to 96.4) | 51.6  (38.7 to 64.2) | 1.72  (1.24 to 2.38) | 0.32  (0.11 to 0.93) | 32.6  (19.5 to 48.0) | 91.7  (77.5 to 98.2) |
|  |  |  |  |  |  |  |  |  |  |
|  | | | | | | | | | |
| ***Cytokines*** | | | | | | | | | |
| ***IL-4 (pg/mL)*** | | | | | | | | | |
| *T2* | 0.697  (0.548 to 0.846) | 0.010 | >2.12 | 72.2  (46.5 to 90.3) | 65.6  (52.7 to 77.1) | 2.10  (1.35 to 3.27) | 0.42  (0.20 to 0.91) | 37.1  (21.5 to 55.1) | 89.4  (76.9 to 96.5) |
|  |  |  |  |  |  |  |  |  |  |
| ***IL-6 (pg/mL)*** | | | | | | | | | |
| *T2* | 0.697  (0.559 to 0.836) | 0.005 | >74.60 | 72.2  (46.5 to 90.3) | 67.2  (54.3 to 78.4) | 2.20  (1.40 to 3.46) | 0.41  (0.19 to 0.89) | 38.2  (22.2 to 56.4) | 89.6  (77.3 to 96.5) |
|  |  |  |  |  |  |  |  |  |  |

All ROC analyses included 82 patients (64 survivors and 18 non-survivors). AUC, Area under the Curve; LR+, Positive Likelihood Ratio; LR-, Negative Likelihood Ratio; PPV, Positive Predictive Value; NPV, Negative Predictive Value; NLR, Neutrophils to Lymphocytes Ratio; CRP, C-reactive protein; IL, interleukin.

**Table E7. Exploratory Spearman correlations between baseline calprotectin and clinical or biological measures at T0**

| **Comparator** | **Spearman ρ** | **Unadjusted p value** | **BH FDR-adjusted q value** | |
| --- | --- | --- | --- | --- |
| **SOFA-2** | 0.224 | 0.043 | 0.156 | |
| ***Biomarkers*** | | | |  |
| **WBC** | 0.424 | <0.001 | **<0.001** | |
| **Neutrophils** | 0.439 | <0.001 | **<0.001** | |
| **Lymphocytes** | -0.041 | 0.712 | 0.757 | |
| **NLR** | 0.355 | 0.001 | **0.008** | |
| **Platelets** | 0.155 | 0.165 | 0.454 | |
| **Creatinine** | 0.134 | 0.232 | 0.463 | |
| **Bilirubin** | -0.139 | 0.212 | 0.463 | |
| **CRP** | 0.281 | 0.011 | **0.046** | |
| **PCT** | 0.071 | 0.528 | 0.726 | |
| ***Cytokines*** | | | | |
| **IL-1α** | -0.159 | 0.154 | 0.454 | |
| **IL-1β** | -0.049 | 0.660 | 0.757 | |
| **IL-2** | 0.040 | 0.722 | 0.757 | |
| **IL-4** | -0.052 | 0.642 | 0.757 | |
| **IL-6** | -0.074 | 0.506 | 0.726 | |
| **IL-8** | -0.080 | 0.473 | 0.726 | |
| **IL-10** | -0.104 | 0.352 | 0.645 | |
| **IFN-γ** | -0.035 | 0.757 | 0.757 | |
| **MCP-1** | -0.136 | 0.222 | 0.463 | |
| **TNF-α** | 0.042 | 0.705 | 0.757 | |
| ***Growth Factors*** | | | | |
| **EGF** | 0.090 | 0.421 | 0.713 | |
| **VEGF** | 0.294 | 0.007 | **0.040** | |

All correlation analyses included the complete cohort of 82 patients. Two-sided p values are reported together with Benjamini-Hochberg false-discovery-rate-adjusted q values calculated across the 22 correlation tests.

SOFA-2, Sequential Organ Failure Assessment 2; WBC, White Blood Cell count; NLR, Neutrophil-to-Lymphocyte Ratio; CRP, C-Reactive Protein; PCT, procalcitonin; IFN-γ, Interferon gamma; MCP-1, Monocyte Chemoattractant Protein-1; EGF, epidermal growth factor; VEGF, vascular endothelial growth factor.

Figure E1. Longitudinal profiles of the remaining routine biomarkers according to 28-day outcome.

Longitudinal distributions of (A) white blood cell count, (B) neutrophil count, (C) lymphocyte count, (D) creatinine, and (E) bilirubin at T0, T1, and T2 in survivors and non-survivors. Trajectory labels report unadjusted p values from the formal group-by-time tests and, where applicable, Benjamini-Hochberg false-discovery-rate-adjusted q values across the 22 trajectory tests. Horizontal brackets identify significant pairwise temporal changes in the overall cohort or a specified outcome group. Between-group labels identify nominal unadjusted survivor-versus-non-survivor differences at the indicated time point and report the corresponding q values from correction across the 63 secondary biomarker-by-time comparisons.

Box plots show every patient-level observation, the median and interquartile range, with whiskers extending from the 10^th^ to the 90^th^ percentile. Where identified in the y-axis title, a logarithmic scale is used for visualization only; original values were plotted and no observations were transformed or excluded. WBC, white blood cell count.

Figure E2. Longitudinal profiles of the remaining cytokines according to 28-day outcome.

Longitudinal distributions of (A) IL-1α, (B) IL-1β, (C) IL-2, (D) IL-8, (E) IL-10, (F) interferon-γ, (G) monocyte chemoattractant protein-1, and (H) tumor necrosis factor-α at T0, T1, and T2 in survivors and non-survivors. Trajectory labels report unadjusted p values from the formal group-by-time tests and, where applicable, Benjamini-Hochberg false-discovery-rate-adjusted q values across the 22 trajectory tests. Horizontal brackets identify significant pairwise temporal changes in the overall cohort or a specified outcome group. Between-group labels identify nominal unadjusted survivor-versus-non-survivor differences at the indicated time point and report the corresponding q values from correction across the 63 secondary biomarker-by-time comparisons.

Box plots show every patient-level observation, the median and interquartile range, with whiskers extending from the 10^th^ to the 90^th^ percentile. Where identified in the y-axis title, a logarithmic scale is used for visualization only; original values were plotted and no observations were transformed or excluded. IFN-γ, interferon gamma; IL, interleukin; MCP-1, monocyte chemoattractant protein-1; TNF-α, tumor necrosis factor alpha.

Figure E3. Longitudinal profiles of growth factors according to 28-day outcome.

Longitudinal distributions of (A) epidermal growth factor (EGF) and (B) vascular endothelial growth factor (VEGF) at T0, T1, and T2 in survivors and non-survivors. Trajectory labels report unadjusted p values from the formal group-by-time tests and, where applicable, Benjamini-Hochberg false-discovery-rate-adjusted q values across the 22 trajectory tests. Horizontal brackets identify significant pairwise temporal changes in the overall cohort or a specified outcome group. Between-group labels identify nominal unadjusted survivor-versus-non-survivor differences at the indicated time point and report the corresponding q values from correction across the 63 secondary biomarker-by-time comparisons.

Box plots show every patient-level observation, the median and interquartile range, with whiskers extending from the 10^th^ to the 90^th^ percentile. Where identified in the y-axis title, a logarithmic scale is used for visualization only; original values were plotted and no observations were transformed or excluded. EGF, epidermal growth factor; VEGF, vascular endothelial growth factor.

Figure E4. Exploratory receiver operating characteristic curves for secondary routine biomarkers.

Receiver operating characteristic curves for discrimination of 28-day mortality by neutrophil count at T1 (A) and T2 (B), lymphocyte count at T2 (C), NLR at T1 (D) and T2 (E), and CRP at T2 (F). AUCs and 95% confidence intervals were estimated using the DeLong method. Orange points indicate the thresholds selected using the Youden index; grey dashed lines indicate chance discrimination. These analyses are descriptive and hypothesis-generating because the corresponding time point-specific between-group comparisons did not remain statistically significant after false-discovery-rate correction. AUC, area under the receiver operating characteristic curve; CI, confidence interval; CRP, C-reactive protein; NLR, neutrophil-to-lymphocyte ratio.

Figure E5. Exploratory receiver operating characteristic curves for selected cytokines.

Receiver operating characteristic curves for discrimination of 28-day mortality by IL-4 at T2 (A) and IL-6 at T2 (B). AUCs and 95% confidence intervals were estimated using the DeLong method. Orange points indicate the thresholds selected using the Youden index; grey dashed lines indicate chance discrimination. These analyses are descriptive and hypothesis-generating because the corresponding time point-specific between-group comparisons did not remain statistically significant after false-discovery-rate correction. AUC, area under the receiver operating characteristic curve; CI, confidence interval; IL, interleukin.

Figure E6. Exploratory correlations between baseline calprotectin and clinical or biological measures.

Color-coded lollipop plot showing Spearman rank-correlation coefficients between calprotectin at T0 and SOFA-2, routine biomarkers, cytokines, and growth factors measured at the same time point. Dot position and color indicate the direction and magnitude of Spearman ρ: blue represents negative correlations, near-white represents values close to zero, and orange represents positive correlations. Larger dots with a dark outline, bold labels, and an asterisk identify correlations that remained statistically significant after Benjamini-Hochberg false-discovery-rate correction across the 22 tests. Exact coefficients and adjusted q values are reported beside each estimate. Correlations were exploratory and were not used for predictor selection or model specification.

CRP, C-reactive protein; EGF, epidermal growth factor; IFN-γ, interferon gamma; MCP-1, monocyte chemoattractant protein-1; NLR, neutrophil-to-lymphocyte ratio; PCT, procalcitonin; SOFA-2, Sequential Organ Failure Assessment 2; VEGF, vascular endothelial growth factor; WBC, white blood cell count.

**REFERENCES**

1.	**Habibzadeh F.** 2024. Data Distribution: Normal or Abnormal? J Korean Med Sci **39:**e35.

2.	**Rochon J, Gondan M, Kieser M.** 2012. To test or not to test: Preliminary assessment of normality when comparing two independent samples. BMC Med Res Methodol **12:**81.

3.	**Rascati KL, Smith MJ, Neilands T.** 2001. Dealing with skewed data: an example using asthma-related costs of medicaid clients. Clin Ther **23:**481-498.

4.	**Sheldon MR, Fillyaw MJ, Thompson WD.** 1996. The use and interpretation of the Friedman test in the analysis of ordinal-scale data in repeated measures designs. Physiother Res Int **1:**221-228.

5.	**Morris L, Galezowski A, Atilla A, Menard J.** 2024. Short-duration peripherally inserted central catheters do not alter viscoelastic parameters in healthy dogs. Can Vet J **65:**692-697.

6.	**Polanco XBJ, Bertasso AS, Silveira HA, Yamamoto de Almeida L, Almeida LKY, da Silva RAB, da Silva LAB, de Rossi A, Nelson-Filho P, Leon JE.** 2021. IgG4-positive plasma cells are more often detected in chronic periapical lesions arising from permanent rather than primary teeth. Int Endod J **54:**682-692.

7.	**Glickman ME, Rao SR, Schultz MR.** 2014. False discovery rate control is a recommended alternative to Bonferroni-type adjustments in health studies. J Clin Epidemiol **67:**850-857.

8.	**Cnaan A, Laird NM, Slasor P.** 1997. Using the general linear mixed model to analyse unbalanced repeated measures and longitudinal data. Stat Med **16:**2349-2380.

9.	**Staffa SJ, Zurakowski D.** 2020. Calculation of Confidence Intervals for Differences in Medians Between Groups and Comparison of Methods. Anesth Analg **130:**542-546.

10.	**DeLong ER, DeLong DM, Clarke-Pearson DL.** 1988. Comparing the areas under two or more correlated receiver operating characteristic curves: a nonparametric approach. Biometrics **44:**837-845.

11.	**Nahm FS.** 2022. Receiver operating characteristic curve: overview and practical use for clinicians. Korean J Anesthesiol **75:**25-36.

12.	**Fluss R, Faraggi D, Reiser B.** 2005. Estimation of the Youden Index and its associated cutoff point. Biom J **47:**458-472.

13.	**Cento V, Carloni S, Sarti R, Bussini L, Asif Z, Morelli P, De Fazio F, Tordato FM, Casana M, Mondatore D, Desai A, Generali E, Pugliese N, Costantini E, Vanoni M, Cecconi M, Aliberti S, Da Rin G, Casari E, Bartoletti M, Voza A.** 2025. Epidemiology and Resistance Profiles of Bacteria Isolated From Blood Samples in Septic Patients at Emergency Department Admission: A 6-Year Single Centre Retrospective Analysis From Northern Italy. J Glob Antimicrob Resist **41:**202-210.

14.	**Todi S, Mehta Y, Zirpe K, Dixit S, Kulkarni AP, Gurav S, Chandankhede SR, Govil D, Saha A, Saha AK, Arunachala S, Borawake K, Bhosale S, Ray S, Gupta R, Kuragayala SD, Samavedam S, Shah M, Hegde A, Gopal P, Ansari AS, Sarkar AK, Pandit R.** 2024. A multicentre prospective registry of one thousand sepsis patients admitted in Indian ICUs: (SEPSIS INDIA) study. Crit Care **28:**375.

15.	**Belati A, Diella L, Bavaro DF, De Santis L, Cotugno S, De Gennaro N, Brindicci G, Maggiore ME, Indraccolo F, Di Gennaro F, Ronga L, Mosca A, Stufano M, Dalfino L, Grasso S, Saracino A.** 2024. Intravenous Fosfomycin as Adjunctive Therapy for Gram-Negative Bacteria Bloodstream Infections: A Propensity Score Adjusted Retrospective Cohort Study. Int J Antimicrob Agents **64:**107247.

16.	**Heinze G, Schemper M.** 2002. A solution to the problem of separation in logistic regression. Stat Med **21:**2409-2419.

17.	**Steyerberg EW, Harrell FE, Jr., Borsboom GJ, Eijkemans MJ, Vergouwe Y, Habbema JD.** 2001. Internal validation of predictive models: efficiency of some procedures for logistic regression analysis. J Clin Epidemiol **54:**774-781.
